# Extraction of Bioactive Compounds from *Larrea cuneifolia* Cav. Using Natural Deep Eutectic Solvents: A Contribution to the Plant Green Extract Validation of Its Pharmacological Potential

**DOI:** 10.3390/plants14071016

**Published:** 2025-03-24

**Authors:** Agostina Conta, Mario Juan Simirgiotis, José Martínez Chamás, María Inés Isla, Iris Catiana Zampini

**Affiliations:** 1 Instituto de Bioprospección y Fisiología Vegetal (INBIOFIV-CONICET-UNT), Facultad de Ciencias Naturales e IML, Universidad Nacional de Tucumán, San Martín 1545, San Miguel de Tucumán 4000, Tucumán, Argentina; agosconta20@gmail.com (A.C.); jmartinezchamas@gmail.com (J.M.C.); misla@csnat.unt.edu.ar (M.I.I.); 2 Instituto de Farmacia, Universidad Austral de Chile, Campus Isla Teja, Valdivia 5090000, Chile; mario.simirgiotis@uach.cl

**Keywords:** green chemistry, medicinal plants, semiarid region, natural deep eutectic solvents (NADESs), UHPLC-MS/MS

## Abstract

A sustainable alternative to replace the use of toxic and non-biodegradable conventional solvents for the extraction of active principles from plants is natural deep eutectic solvents (NADESs). *Larrea cuneifolia* Cav. (Zygophyllaceae) is a plant widely distributed in semiarid areas of western Argentina. Several studies validate its popular medicinal use by demonstrating its biological activities such as antibacterial, antifungal, antioxidant, anti-inflammatory, and anticarcinogenic properties, among others. The aim of this work was to compare the bioactive compounds and the in vitro antioxidant and antibacterial activity of *L. cuneifolia* extracts using non-conventional vs. conventional solvents. Aqueous, ethanolic, and four NADES extracts were prepared. The extracts were phytochemically characterized, and extracted compounds were identified by UHPLC-MS/MS. Antioxidant activity was determined by evaluating the hydrogen peroxide and free radical scavenging capacity using ABTS^•+^. The antibacterial activity of the extracts and NADESs was evaluated against Gram-positive and Gram-negative multidrug-resistant strains. The extracts of *L. cuneifolia* presented a variable content of total phenolic compounds between 4163.4 and 24,371.63 µg GAE/mL. Phenolic acids, flavonoid glycosides, flavanones, flavones, flavonols, alkaloids, lignans (nordihydroguaiaretic acid and its derivatives), and other compounds were tentatively identified in extracts of *L. cuneifolia* obtained with conventional and non-conventional solvents. A heatmap cluster and a bubble plot were created to compare the diversity and relative abundance of identified compounds, and the extracts were classified into two major groups. All extracts were able to scavenge > 40% of hydrogen peroxide and the ABTS radical cation (ABTS^•+^) (CD_50_ = 3.15–5.13 µg GAE/mL). The LAS extract exhibited the highest bacterial growth inhibition (MIC = 75–37.5 µg GAE/mL). In conclusion, the results show that NADESs represent a sustainable alternative for the extraction of compounds with antioxidant and antibacterial activity and could therefore replace traditional solvents in the pharmaceutical, cosmetic, or food industries.

## 1. Introduction

Plants can produce a great diversity of organic compounds in response to their environment to achieve successful growth and continuity in the species. These are secondary metabolites, and their production may be associated in response to biotic or abiotic stress. Beyond their intrinsic value to the plant, many secondary metabolites are commercially valuable as several are known to exhibit medicinal properties, making them pharmacologically active compounds important for human health [1]. There is an urgent and increasing demand for plant-based medicine in developing countries because more than 80% of the population relies on herbal-based treatment for necessary healthcare [2].

*Larrea cuneifolia* Cav. (Zygophyllaceae) is a xerophytic evergreen shrub found in arid/semiarid areas of Argentina that is used in traditional medicine to treat different ailments. According to several ethnobotanical studies, it is widely employed by local communities as an anti-rheumatism, anti-inflammatory, antibacterial, antifungal, and anticancer agent. Previous studies have demonstrated the antibacterial activity of ethanolic extracts of *L. cuneifolia* against several multidrug-resistant Gram-negative bacteria obtained from clinical isolates [3] as well as against bacterial strains of veterinary interest [4]. Antioxidant [5], anti-inflammatory [6,7,8], antifungal [5,9], and anticancer [10] effects of *L. cuneifolia* extracts and herbal mixtures including *L. cuneifolia* were also demonstrated.

Some phytochemical studies of *L. cuneifolia* reported the presence of phenolic compounds, including flavonoids such as quercetin, apigenin, and kaempferol derivatives, as well as lignans, cyclolignans, and epoxylignans, with nordihydroguaiaretic acid (NDGA) representing the chemical marker of the species and being the most abundant [5,7,11]. These compounds, both flavonoids and lignans, have received a lot of attention due to their antimicrobial and antioxidant properties, which are strongly related to the prevention of cancer, inflammatory disorders, and cardiovascular conditions [12]. In this sense, it is important to achieve an efficient and selective extraction of bioactive compounds present in plant matrices. For this purpose, the choice of an appropriate solvent is crucial to achieve a good extraction yield. In terms of the effectiveness of phenolic compound extraction, using conventional solvents like methanol, ethyl acetate, or chloroform makes sense. However, these substances have negative effects on the environment and human health which are not insignificant. One key concern with these solvents is that they can be toxic, carcinogenic, non-biodegradable, or require an additional step in the bioproduct manufacturing process to remove them. Furthermore, traditional solvent extraction techniques are not cost-effective as they require an excessive amount of energy, time, and solvents [13,14].

Green chemistry was first introduced in the 1990s, and it outlines twelve principles that provide the framework for the sustainable design of safer chemicals and chemical processes [15]. In this way, in recent years, there has been an increasing interest in the search for environmentally friendly and safe solvents for the extraction of bioactive compounds to replace organic solvents hazardous to human health and the environment.

In 2003, a new type of solvent based on eutectic mixtures called deep eutectic solvents (DESs) was introduced [16]. The term “eutectic” (“eu”: easy; “teksis”: melting) refers to a mixture of components that in certain ratios exhibit a significantly lower melting point than their individual components, mainly due to the formation of intermolecular hydrogen bonds and Van der Walls interactions. The term natural deep eutectic solvent (NADES) refers to eutectic mixtures constituted by two or more natural metabolites present in the cells of all living organisms, such as organic acids (lactic acid, malic acid, citric acid, etc.), sugars (fructose, sucrose, glucose, etc.), alcohols, amino acids, urea, choline derivatives, and usually also a certain mole ratio of water, which can be found in liquid state at room temperature [17]. Structurally, NADESs are mixtures formed by different combinations of hydrogen bond donors (HBDs) and hydrogen bond acceptors (HBAs), which allow these to be design solvents, with tunable physicochemical properties in accordance with the objectives of each researcher [18].

In this regard, NADESs represent an interesting sustainable alternative to the use of toxic organic solvents since they offer numerous application advantages as they are biodegradable, easy to prepare, non-toxic, eco-friendly, free of waste, and biocompatible, and could also be used as phytotherapeutic agents, fully following the principles of green chemistry [19]. In this context, NADESs present great potential as alternative solvents for the extraction of bioactive plant-derived compounds. Due to their properties, they could improve the extraction efficiency, solubility, stability, bioactivity, and bioavailability of plant bioactive compounds [13,20].

In this sense, the aim of this work is to compare the extraction of bioactive compounds from *L. cuneifolia* using NADES-based systems as novel and sustainable green solvents, vs. conventional solvents, to evaluate the biological activity (antioxidant and antibacterial) of the obtained extracts.

## 2. Results

### 2.1. NADES Preparation

In general, the extraction yield of different types of phytochemical compounds present in plants can potentially be influenced by both the extraction conditions employed (extraction technique, temperature, and solid/liquid ratio) and the physicochemical properties of the solvent used. Furthermore, the chemical composition of NADESs is crucial for their ability to extract compounds, as it determines properties such as polarity, physicochemical interactions, solubility, and viscosity. Consequently, to select the most appropriate NADES for the extraction of bioactive compounds from *L. cuneifolia*, two classes of NADESs were used: (1) organic-acid-based (LAS, CAP, and SALA); (2) sugar-based (FGS) (Table 1). In addition, conventional solvents (distilled water and 70% ethanol) were used for comparative purposes.

Regarding the composition of NADESs, sugars, alcohols, and organic acids are all components that are hydrogen bond donors and acceptors, which is believed to be the basis for the complexation of solids giving rise to liquids with a supramolecular structure. In fact, NADESs are like liquid crystals in which all molecules are arranged through hydrogen bonds and other physical intermolecular bonding forces [18].

As can be seen in Table 1, in the preparation of some NADESs, the addition of water was necessary in the initial synthesis of the NADES or once the mixture was formed. A disadvantage of NADESs, with respect to conventional solvents, is their high viscosity, making mass transfer difficult. This is the reason why some authors propose adding a small percentage of water (% *w*/*w*) to reduce the preparation time and temperature and decrease its viscosity to maintain or increase the extraction efficiency of bioactive compounds. However, a large dilution of NADESs with water result in the loss of existing hydrogen bonds and, consequently, the disappearance of the structure of the NADES, and a simple aqueous solution of the individual components could be obtained. Dilutions below 50% generally do not lead to the breakdown of the eutectic system into its individual components [21,22]. Therefore, water ratios compatible with the stability of liquid NADESs at room temperature were used. In fact, the initial synthesis of NADES-FGS was not possible without the addition of water, i.e., water is an integral part of the supramolecular structure of NADESs [18], the same for NADES-SALA. In contrast, for NADES-LAS, the addition of 15% *w*/*w* water was necessary once the mixture was formed (Table 1). Thus, all prepared NADESs proved to be stable and transparent liquids that do not precipitate during preparation, extraction, or analysis.

Regarding the solvents’ polarities, NADES-FGS exhibits polarity equal to water (ENR = 48.21 kcal/mol) [18], while NADES-CAP exhibits polarity comparable to ethanol 70% [23]. On the other hand, NADES-LAS was selected from the literature, as it has been shown to present good properties in the extraction of phenolic compounds [24], and NADES-SALA [25] was incorporated to compare the effect of water as a component of the eutectic system, since in the case of NADES-LAS, water was added for diluent purposes once the NADES was obtained.

**Table 1:** Main characteristics of used natural deep eutectic solvents (NADESs).

NADES	Eutectic Mixture	Molar Ratio	Water Content (% *w*/*w*)	pH	Conditions	Observations/Visual Aspect *	Ref.
LAS	Lactic acid–saccharose	4:1	15	4	60 min 40 °C	Transparent liquid	[24]
CAP	Citric acid–propylen glycol	1:4	-	5	20 min 40 °C	Transparent viscous liquid	[23]
FGS	Fructose–glucose–saccharose–water	1:1:1:11	-	6	20 min 40 °C	Transparent liquid	[18,26]
SALA	Lactic acid–saccharose–water	1:5:7	-	1	20 min 40 °C	Transparent liquid	[25]

* At room temperature.

### 2.2. Phytochemical Screening

In order to compare the extraction performance of NADESs and conventional solvents, the content of different phytochemicals groups, i.e., total phenolic compounds, flavonoids, and alkaloids, was determined by spectrophotometric methods.

**Figure 1:**
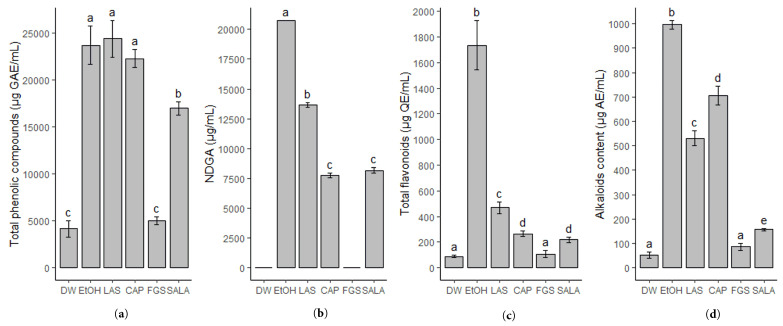
Phytochemical screening of *Larrea cuneifolia* Cav. extracts. (**a**) Total phenolic compounds (µg GAE/mL); (**b**) NDGA content (µg/mL); (**c**) total flavonoids content (µg QE/mL); (**d**) alkaloid content (µg AE/mL). GAE: gallic acid equivalent; QE: quercetin equivalent; AE: apomorphine chlorhydrate equivalent. (DW: aqueous extract; EtOH: ethanolic extract; different extracts obtained with NADESs include: LAS: lactic acid–saccharose; CAP: citric acid–propylene glycol; SALA: lactic acid–saccharose–water; FGS: fructose–glucose–saccharose–water.). Different letters above the bars represent statistically significant differences at *p* ≤ 0.05, according to Tukey’s test.

The extracts of *L. cuneifolia* present a variable content of total phenolic compounds between 4163.4 and 24,371.63 µg GAE/mL. The results are shown in Figure 1a. The extracts obtained with the non-conventional solvents NADES-LAS and NADES-CAP showed the highest content of total phenolic compounds without significant differences compared to the extract obtained with 70% ethanol. The sugar-based NADES-FGS is the solvent system that shows the lowest extraction yield of total phenolic compounds without statistically significant differences compared to distilled water. The SALA extract showed a lower content of phenolic compounds compared to the LAS extract, even though the solvent also consists of lactic acid and saccharose. The similarities in polarity of the different solvents used are reflected in the extraction yield of phenolic compounds.

Furthermore, the chemical marker of *L. cuneifolia*, the lignan nordihydroguaiaretic acid (NDGA), was quantified using HPLC-DAD (Figure 1b). It is noteworthy that several studies have reported this compound to be responsible for various biological activities in *Larrea* spp. The most selective non-conventional solvent for NDGA extraction proves to be NADES-LAS (13,496.3 µg/mL). CAP and SALA extracts also showed good NDGA extraction performance with no significant differences between them. However, the DW and FGS extracts were unable to extract NDGA.

The flavonoid content was also evaluated considering that numerous of these compounds present biological activities and could act as antioxidants and antibacterial [27], and several flavonoids were previously reported for *L. cuneifolia*. Regarding this, NADESs based on lactic acid (LAS) prove to be the best eutectic solvent for flavonoid extraction (469.5 µg QE/mL). However, the flavonoid extraction yield of all the NADESs employed is significantly lower than 70% ethanol (1582.7 µg QE/mL). Both water and FGS are the solvents that show the lowest flavonoid extraction efficiency (Figure 1c). All extracts remained stable in terms of total phenolic compounds and total flavonoid content after 3 and 6 months.

**Figure 2:**
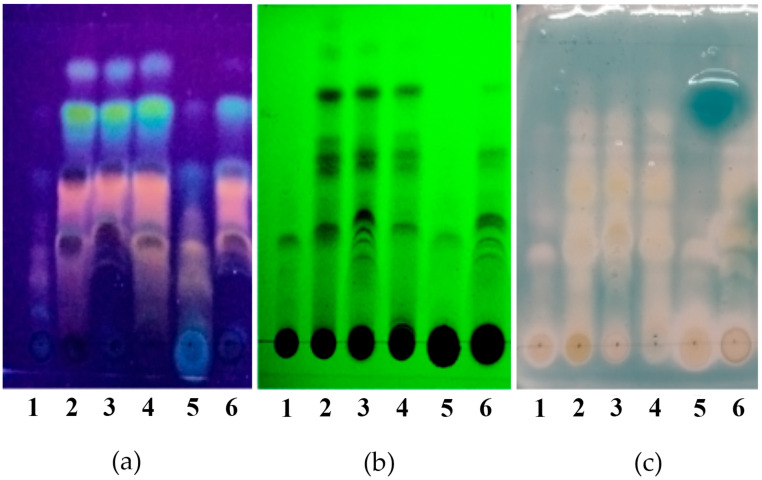
Thin-layer chromatography (TLC) of *L. cuneifolia* extracts. (**a**) Separated components visualized under UV light at 365 nm + NP/PEG reagent (NP 1% diphenylborinic acid aminoethyl ester in methanol; PEG: poly-ethylene glycol); (**b**) separated components visualized under UV light at 254 nm; (**c**) autographic method for the detection of antioxidant activity using ABTS^•+^ of *L. cuneifolia* extracts. **1**: Aqueous extract (DW); **2**: ethanolic extract (EtOH); different extracts obtained with NADESs include: **3**: lactic acid–saccharose (LAS); **4**: citric acid–propylene glycol (CAP); **5**: fructose–glucose–saccharose–water (FGS); **6**: lactic acid–saccharose–water (SALA).

Phenolic compound profiles were also analyzed by thin-layer chromatography; in all the extracts, a great diversity of bands is observed that reveal light blue, green, yellow, and orange colors with the NP/PEG reagent, which would indicate the presence of flavonoids and phenolic compounds of the phenolic acid type (Figure 2). Additionally, the same profile of compounds is obtained with organic-acid-based NADESs as with ethanolic extracts. Similar profiles can be seen for FGS and aqueous extracts, both of which show a high concentration of polar compounds.

In previous work, the presence of alkaloids in *L. cuneifolia* was reported, so the alkaloid content in *L. cuneifolia* conventional and non-conventional extracts was determined by spectrophotometry. The results are shown in Figure 1d. NADES-CAP shows the highest extraction efficiency, followed by LAS and SALA (706.67 µg AE/mL; 530.77 µg AE/mL; 156.41 µg AE/mL, respectively). Nevertheless, the ethanolic extract exhibits the best alkaloid extraction yield (996.92 µg AE/mL). Again, DW and FGS extracts were demonstrated to be the poorest in terms of alkaloid compounds with similar content (51.79 µg AE/mL and 85.64 µg AE/mL).

### 2.3. UHPLC-MS/MS

In the last few years, there have been studies on the chemical composition of *L. cuneifolia*, with the discovery of novel chemical compounds identified in extracts obtained through conventional solvents like ethanol, methanol, water, and others. The chemical identification of compounds in extracts obtained using NADESs has been underexplored.

**Figure 3:**
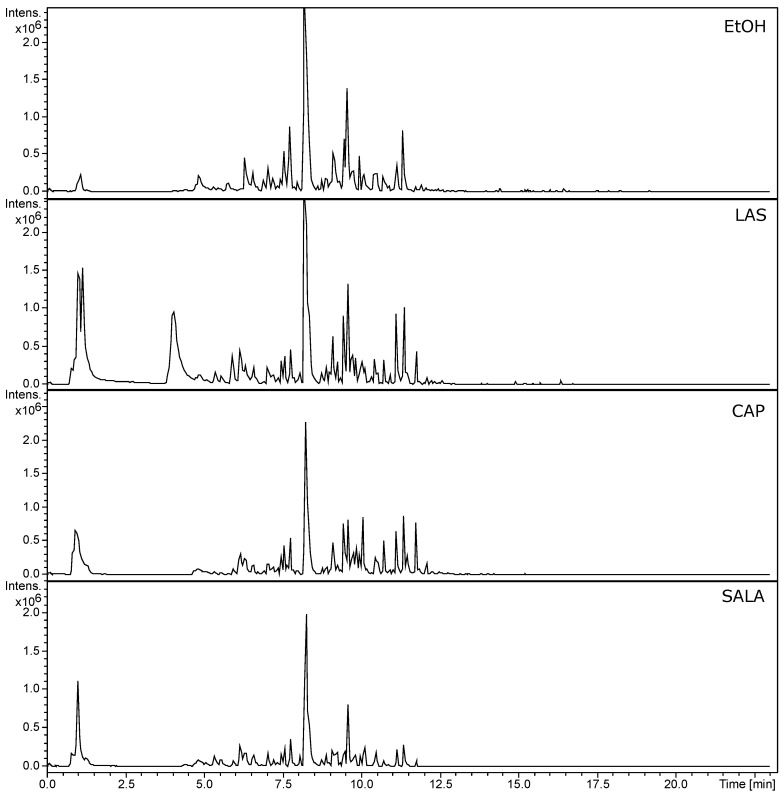

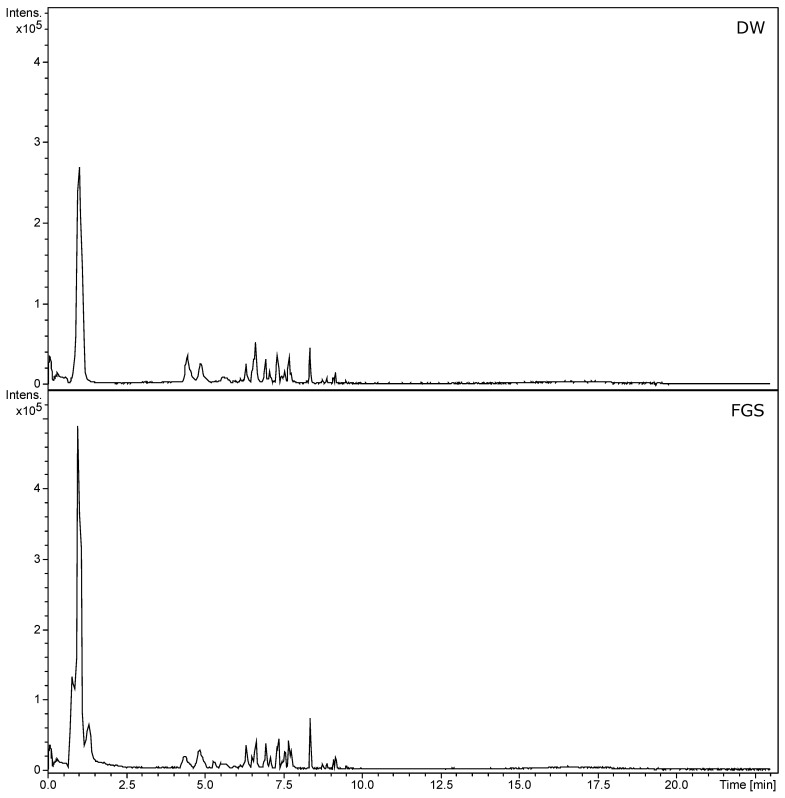
UHPLC-PDA-ESI-QT-MS/MS chromatograms (TIC, total ion current) of *L. cuneifolia* extracts employing different solvents under ion negative mode. (DW: aqueous extract; EtOH: ethanolic extract; different extracts obtained with NADESs: LAS: lactic acid–saccharose; CAP: citric acid–propylene glycol; SALA: lactic acid–saccharose–water; FGS: fructose–glucose–saccharose–water.)

In this study, we analyzed the chemical profiles obtained by UHPLC of all *L. cuneifolia* extracts using non-conventional and conventional solvents. In Table S1, the tentatively identified compounds are listed. UHPLC-MS-MS chromatograms (TIC, total ion current) are shown in Figure 3. The profiles exhibit notable similarities between the ethanolic extract (EtOH) and the organic-acid-based NADESs, as well as between the DW extract and the FGS. The prominent peak corresponds to NDGA (compound **68** in Table S1; Rt: 8.25 min in negative mode), the chemical marker of *Larrea* spp. Additionally, the EtOH, LAS, CAP, and SALA extracts demonstrate a greater diversity of compounds compared to DW and FGS. EtOH extract shows higher peak intensity, followed by LAS > CAP > SALA.

Using UHPLC-PDA-ESI-QT-MS/MS analyses in positive and negative modes, 113 compounds were tentatively identified, including phenolic acids, flavonoid glycosides, flavanones, flavones, flavonols, NDGA, NDGA derivatives, and alkaloids in the extracts of *L. cuneifolia* obtained with different solvents.

Among 113 compounds obtained from the analysis (Table S1), 48 compounds were detected in positive ionization mode, and 65 analytes were detected in negative ionization mode. This suggests that to comprehensively profile the metabolome of *L. cuneifolia* extracts, both positive and negative ionization modes are necessary.

According to the signal intensity values of each compound provided by the metabolomic analysis, which are directly proportional to the detected analyte relative quantity, it can be determined that the compounds found in higher relative abundance include NDGA, 5-(3,4-dihydroxyphenyl)-6,7-dimethyl-5,6,7,8-tetrahydronaphthalene-2,3-diol, heminordihydroguayaretic acid (HNDGA), 3,6-dimethoxyapigenin, tricin, pachypodol, isorhamnetin, piscidic acid, malabaricano, and erucamide (Table S1).

**Figure 4:**
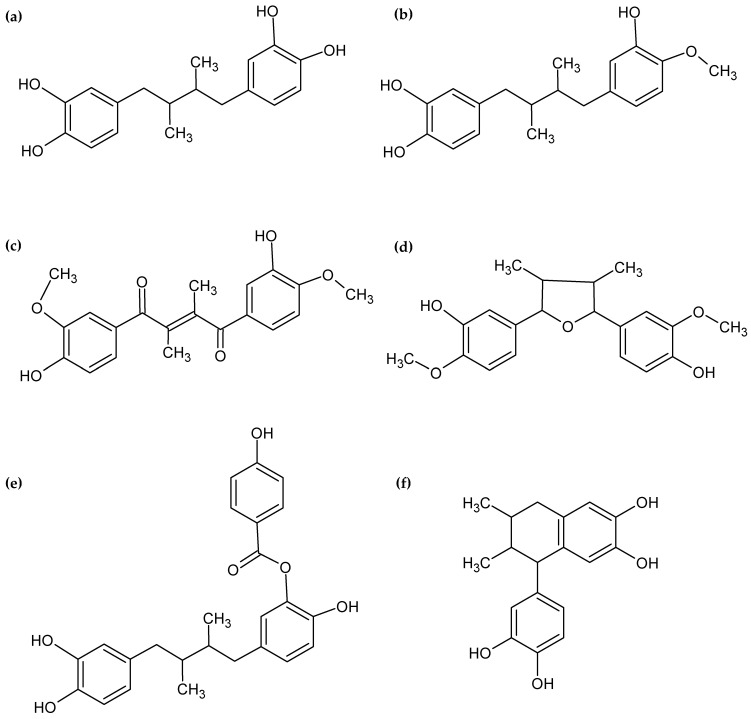
Chemical structures of NDGA and related compounds. (**a**) NDGA, (**b**) HNDGA, (**c**) (E)-1,4-bis(4-hydroxy-3-methoxyphenyl)-2,3-dimethylbut-2-ene-1,4-dione, (**d**) malabaricano, (**e**) [5-[4-(3,4-dihydroxyphenyl)-2,3-dimethylbutyl]-2-hydroxyphenyl] 4-hydroxybenzoate, and (**f**) 5-(3,4-dihydroxyphenyl)-6,7-dimethyl-5,6,7,8-tetrahydronaphthalene-2,3-diol.

Six lignans were tentatively identified in the analysis (NDGA, HNDGA, malabaricano, (E)-1,4-bis(4-hydroxy-3-methoxyphenyl)-2,3-dimethylbut-2-ene-1,4-dione, 5-(3,4-dihydroxyphenyl)-6,7-dimethyl-5,6,7,8-tetrahydronaphthalene-2,3-diol, and [5-[4-(3,4-dihydroxyphenyl)-2,3-dimethylbutyl]-2-hydroxyphenyl] 4-hydroxybenzoate). NDGA has been previously reported in other *Larrea* spp., since this is the chemical marker of these species [6,7,8,10,28]. Additionally, the HNDGA, also known as 3′-O-methyl-nordihydroguayaretic acid, which exhibits a slight chemical modification, was also previously reported by [6,7]. Other compounds related to NDGA were also identified. The compound identified as 4,4′-dihydroxy-3,3′-dimethoxy-7,7′-epoxylignan, also known as malabaricano, was found to be similar to epoxylignans reported in *L. cuneifolia* by [7,28] with different substitutions. Furthermore, malabaricano closely resembles a cyclized analog of this NDGA derivative reported by [29]. On the other hand, the compound identified as (E)-1,4-bis(4-hydroxy-3-methoxyphenyl)-2,3-dimethylbut-2-ene-1,4-dione is related to NDGA and shows similarity to an analog of NDGA reported by [29]. The cyclolignan 5-(3,4-dihydroxyphenyl)-6,7-dimethyl-5,6,7,8-tetrahydronaphthalene-2,3-diol identified in the analysis exhibits similarities to a cyclic analog of NDGA reported by [30]. Additionally, this cyclolignan shows similarity to cyclolignans previously reported in *L. cuneifolia* by [7]. The chemical structures of NDGA and related compounds identified in this study are illustrated in Figure 4.

In addition, a total of 11 phenolic acids and 33 flavonoids were also tentatively identified, including several flavonoid glycosides, which are commonly found in plants. Glycosylation serves as an effective method to alter the water solubility and stability of flavonoids, thereby influencing their biological activities, such as antioxidant, immunomodulatory, and anticancer activities, and it typically affects in vivo metabolism and absorption. Furthermore, glycosylation enhances the bioavailability of flavonoid aglycones by binding to glucose transporters [31]. Consequently, the extraction of these compounds is of significant interest. This study identified seven glycosylated flavonoids, among which several were found to be linked with mono-, di-, or trisaccharides through either -O or -C bonds (compounds **7**, **9**, **10**, **14**, **15**, **27**, and **30** in Table S1). The DW and FGS extracts contained only C-glycosylated flavonoids (compounds **9** and **10**), while the LAS extract exclusively presented O-glycosylated flavonoids (compounds **14**, **15**, **27**, and **30**). The EtOH, CAP, and SALA extracts showed both O-glycosylated and C-glycosylated flavonoids. The extracts obtained with lactic-acid-based NADESs (LAS and SALA) exhibited the greatest diversity of glycosylated flavonoids.

The tentative identification of naringenin, isorhamnetin, and kaempferide in *L. cuneifolia* extracts is in agreement with prior phytochemical research [11,28].

In the present investigation, two alkaloids in *L. cuneifolia* extracts were identified, i.e., salsolinol and trigonelline hydrochloride. The presence of alkaloids in *L. cuneifolia* was previously reported by [32].

**Figure 5:**
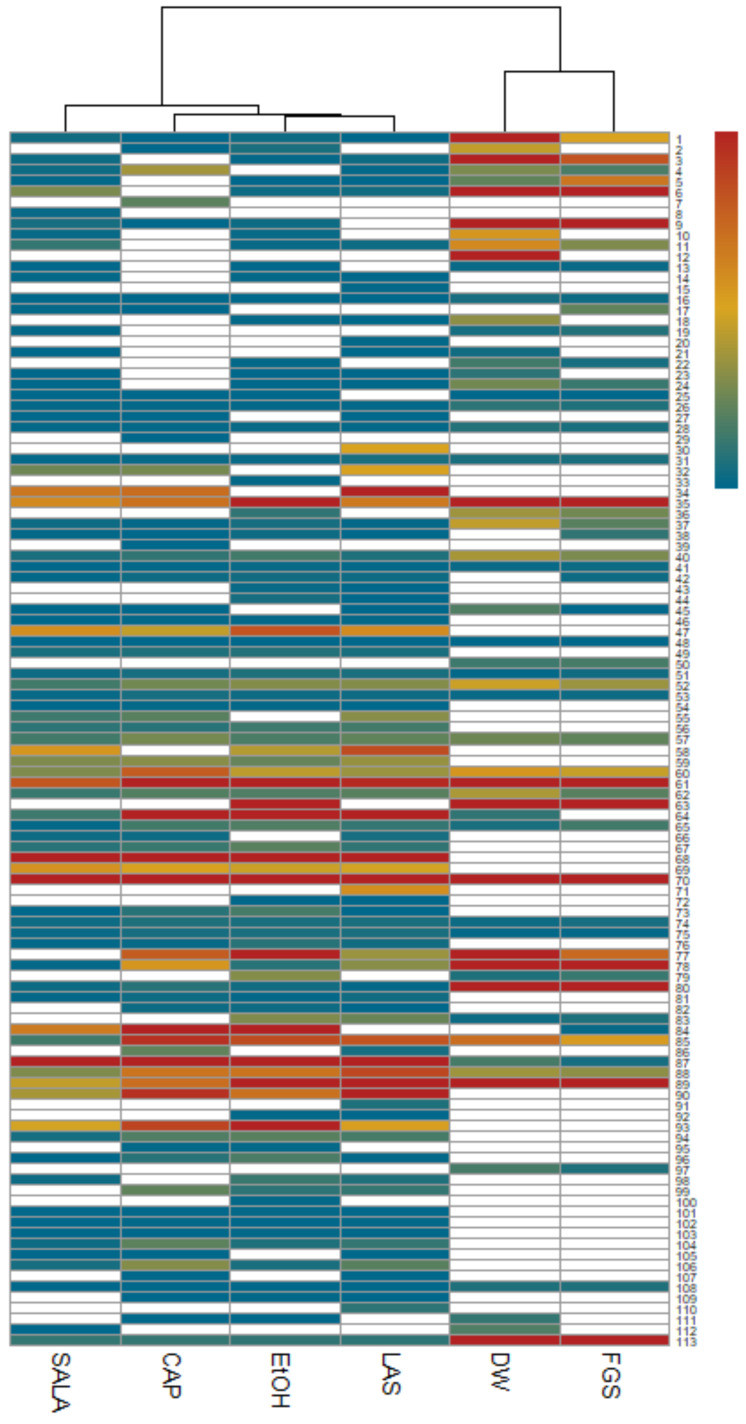
Heatmap representing the relative abundances of compounds across different extracts of *L. cuneifolia*. (DW: aqueous extract; EtOH: ethanolic extract; different extracts obtained with NADESs: LAS: lactic acid–saccharose; CAP: citric acid–propylene glycol; SALA: lactic acid–saccharose–water; FGS: fructose–glucose–saccharose–water.)

A heatmap was generated where the rows represent compounds identified by UHPLC-MS/MS and the matrix columns represent the extracts obtained with different solvents (Figure 5). The results are visualized using a color scale, with red indicating an increase and blue indicating a decrease in compound values across the various extracts. The values inputted in the analysis correspond to the signal intensity values of each compound provided by metabolomic analysis, which are directly proportional to the detected analyte quantity. The blank cells correspond to the absence of a signal for that compound. Subsequently, these values were normalized to construct the heatmap. Moreover, the cluster analysis based on Euclidean distance not only facilitated the grouping of extracts according to their relative abundances but also revealed distinct patterns within these clusters. Notably, two primary clusters emerged: cluster 1, which included DW and FGS extracts, and cluster 2, comprising EtOH extract and those characterized by non-conventional solvents that were organic-acid-based (LAS, CAP, and SALA). Within cluster 2, a more detailed examination unraveled a notable similarity between EtOH-LAS extracts and, interestingly, a pronounced resemblance of these extracts to the CAP extract. The heatmap further clearly illustrates a greater diversity of compounds in the EtOH extract and extracts obtained with NADESs based on organic acids compared to the DW and FGS extracts.

**Figure 6:**
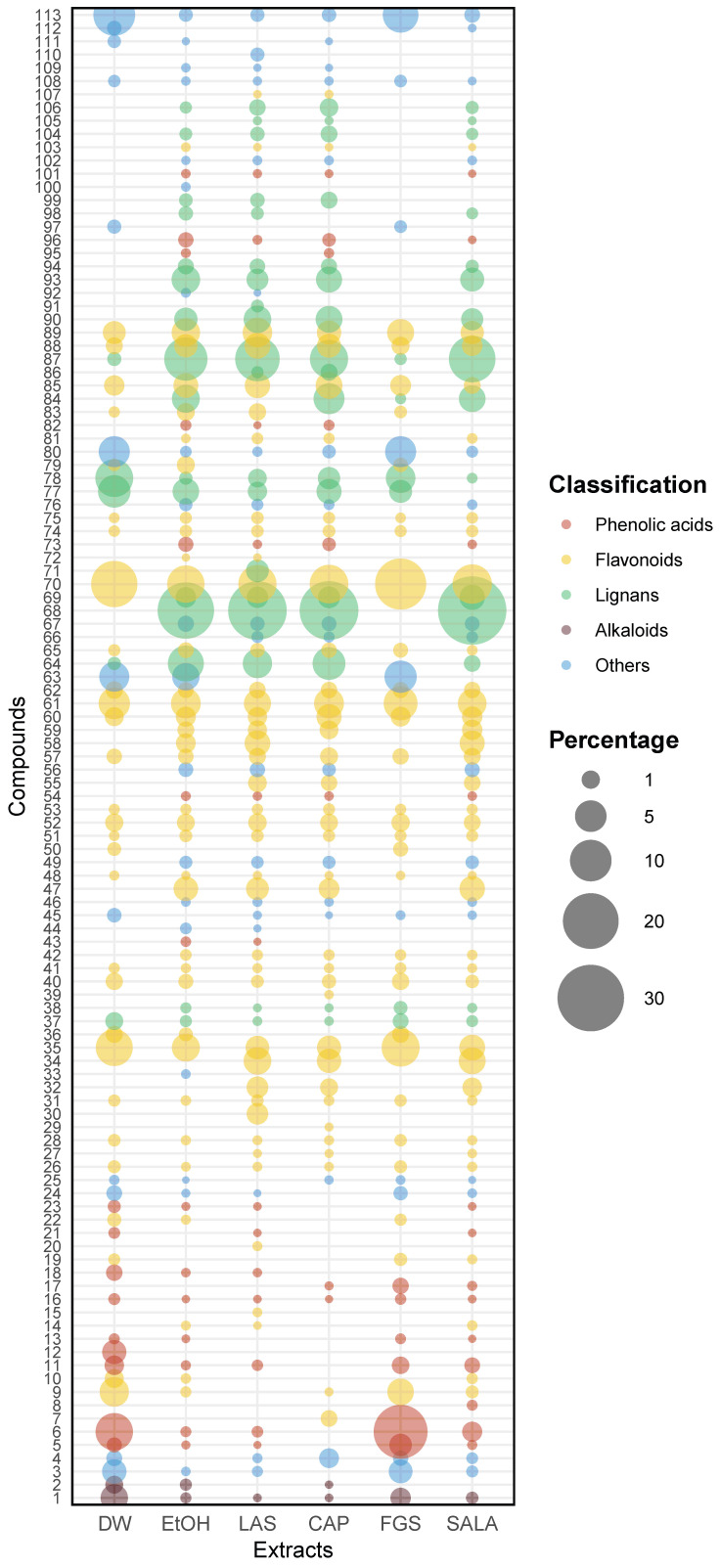
Bubble plot illustrating relative abundance and diversity of identified compounds across various extracts of *L. cuneifolia*. Bubble size represents relative abundance (%), while color indicates compound group classification. (DW: aqueous extract; EtOH: ethanolic extract; different extracts obtained with NADESs: LAS: lactic acid–saccharose; CAP: citric acid–propylene glycol; SALA: lactic acid–saccharose–water; FGS: fructose–glucose–saccharose–water.)

To enrich the analysis of the heatmap, we generated a bubble chart that provides a visual representation of the data (Figure 6). This additional visualization offers valuable insights into the diversity and relative abundances of the tentatively identified compounds by UHPLC-MS/MS across different extracts and allows us to identify patterns, trends, and relationships that may not be immediately apparent from the heatmap alone, offering a multidimensional perspective by incorporating the size and colors of the bubbles as additional variables. In this plot, each bubble represents a specific compound present in different extracts, and the size of the bubble indicates the relative abundance of that compound in terms of percentages. Additionally, the color of each bubble represents the classification of the compound, indicating the broader class to which it belongs. Together, these visualizations enable a more intuitive and comprehensive chemical profile analysis of *L. cuneifolia* extracts, making UHPLC-MS/MS data more accessible and interpretable. In the bubble plot, we can observe a similar pattern to that in the heatmap, with similarities in diversity and relative abundances between DW-FGS extracts and EtOH and organic-acid-based NADES extracts.

Considering the abundance of flavonoids and phenolic acids, the DW and FGS extracts demonstrated higher relative levels but lower diversity compared to the EtOH extract and those obtained using organic-acid-based NADESs, as depicted in the bubble plot. Notably, the DW and FGS extracts were particularly enriched in flavonoids, constituting approximately 43–48% of the relative abundance. Furthermore, these extracts displayed elevated relative proportions of phenolic acids in comparison to the other extracts (13–22%).

The FGS and DW extracts exhibited higher percentages of 3,6-dimethoxyapigenin, isorhamnetin, and piscidic acid compared to the other extracts, in terms of relative abundance. Notably, in the FGS extract, these compounds collectively account for up to 50% of the total compounds. In contrast, the extracts from LAS, CAP, SALA, and EtOH exhibited higher percentages of NDGA and its derivatives, along with flavonoids, making them notably diverse and abundant in relative terms, as illustrated in the bubble plot. These extracts were enriched in NDGA and flavonoids, comprising over 50% lignans and more than 35% flavonoids. Additionally, they displayed greater diversity of these compounds. Specifically, EtOH, LAS, CAP, and SALA extracts accounted for 92%, 96.8%, 95.6%, and 94.6%, respectively, in terms of the combined percentage of flavonoids and lignans.

Furthermore, in addition to these major classes of secondary metabolites, other compounds were identified as mentioned in Figure 6 under the category ‘Others’. This category encompasses organic acids such as D-(−) quinic acid, phenylacetic acid, aldehydes, lactones, terpenes, diarylheptanoids, chlorophyll derivatives, and plant hormones such as gibberellin A7 (Table S1). Although they all are not typical secondary metabolites, these compounds were considered in the analysis due to their reported numerous biological activities.

### 2.4. Antioxidant Activity

The polarity and solubility of the extracted components, structure (number and position of hydroxyl groups), type of free radicals, and their mechanisms of interaction with other components can all affect the antioxidant capacity of an extract. In addition, the type of solvent used to extract antioxidant chemicals can be especially important.

The antioxidant activity of the extracts obtained from *L. cuneifolia* was determined by evaluating the ABTS^•+^ scavenging capacity and hydrogen peroxide scavenging capacity (HPS).

**Figure 7:**
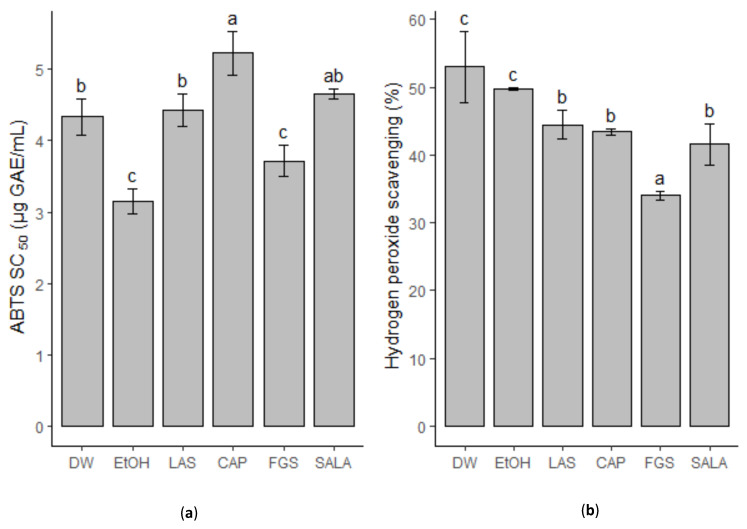
Antioxidant activity of *L. cuneifolia* extracts. (**a**) Scavenging ABTS^•+^; SC_50_ (µg GAE/mL) values correspond to the concentration capable of scavenging 50% of the free radicals; (**b**) hydrogen peroxide scavenging (%). (DW: aqueous extract; EtOH: ethanolic extract; different extracts obtained with NADESs: LAS: lactic acid–saccharose; CAP: citric acid–propylene glycol; SALA: lactic acid–saccharose–water; FGS: fructose–glucose–saccharose–water.). Different letters above the bars represent statistically significant differences at *p* ≤ 0.05, according to Tukey’s test.

The antioxidant capacity of *L. cuneifolia* extracts with different solvents is presented in Figure 7. All extracts were able to scavenge the ABTS^•+^, presenting SC_50_ values between 3.15 and 5.13 µg GAE/mL, being the extract obtained with a sugar-based system that had the most active non-conventional extracts. The extracts obtained by NADESs based on sugars (FGS) presented antioxidant potential comparable to the EtOH extract with an SC_50_ value of 3.71 µg GAE/mL. LAS and SALA extracts do not show significant differences from the DW extract.

On the other hand, the antioxidant activity of the extracts was further assessed qualitatively by autography assay to determine if the compounds separated by thin-layer chromatography preserve their capacity to scavenge the ABTS^•+^. The presence of several inhibitory bands which coincide with light blue, green, and yellow bands are observed for the different extracts, suggesting that the extracts would contain multiple compounds with antioxidant activity, some of which may be phenolic compounds like phenolic acids (light blue bands) and flavonoids (green and yellow bands) (Figure 2).

Under biological conditions, hydrogen peroxide is a weak oxidant that can oxidize thiol groups of proteins or polyunsaturated acyl groups in lipids. However, the reaction of hydrogen peroxide with iron (Fe^2+^) or O_2_^•−^ generates a more reactive radical, the hydroxyl radical (HO^•^). Therefore, it is important to evaluate hydrogen peroxide scavenging as another antioxidant mechanism. Regarding this, all extracts were able to scavenge between 40 and 50% of hydrogen peroxide (Figure 7b). In this case, the FGS extract has the lowest percentage of hydrogen peroxide scavenging (34%).

It is important to emphasize that the antioxidant activity measured corresponds completely to the compounds extracted from *L. cuneifolia*, since each pure solvent was controlled. Therefore, any potential action associated with NADESs is excluded at the dilutions tested.

**Figure 8:**
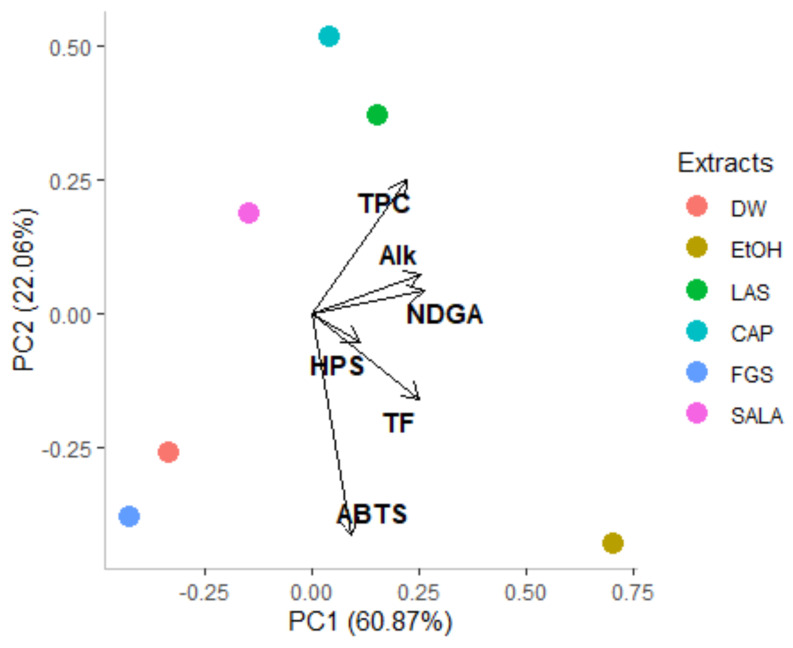
Principal component loading plot and scores of principal component analysis (PCA) of total phenolic compounds (TPC), total flavonoids (TF), nordihydroguaiaretic acid (NDGA), alkaloids content (Alk), scavenging capacity of the ABTS radical cation (ABTS), and hydrogen peroxide scavenging capacity (HPS) of different extracts obtained from *L. cuneifolia*. (DW: aqueous extract; EtOH: ethanolic extract; different extracts obtained with NADESs: LAS: lactic acid–saccharose; CAP: citric acid–propylene glycol; SALA: lactic acid–saccharose–water; FGS: fructose–glucose–saccharose–water).

An exploratory principal components analysis (PCA) was performed to determine the relationships between the solvents used and to detect potential sample grouping according to their chemical composition and biological activity. The PCA offers essential insights into the influence of individual variables on the overall variability that a heatmap cannot offer on its own. The PCA score plot (PC1 vs. PC2) is reported in Figure 8. The first two components synthesize a cumulative variance of 82.93%. The first component explains 60.87% and the second component 22.06% of the overall variance (Table 2). PC1 is positively correlated with all variables studied (Table 2), while PC2 is positively correlated with total phenolic, NDGA, and alkaloid content and is negatively correlated with total flavonoid content and antioxidant activity.

TPC, TF, NDGA, and alkaloid content are the variables most strongly correlated with PC1, indicating that they play a key role in explaining the overall variability among the samples. This suggests that extracts with higher concentrations of phenolic compounds, flavonoids, NDGA, and alkaloids will cluster on the positive side of PC1. The PCA further highlights that TPC, TF, and NDGA are positively correlated with antioxidant activity along PC1, emphasizing the importance of these compounds in driving the variation summarized by the PC1. Thus, extracts with higher PC1 scores tend to have higher concentrations of these compounds, which are associated with greater antioxidant activity.

PCA also clusters the samples based on overall variance, whereas a heatmap groups extracts by Euclidean distance, providing a different perspective on relationships between variables and extracts. For example, the results show that LAS, CAP, and SALA are grouped into a cluster in the upper side of the score plot, which corresponds to the acid-based NADES systems. LAS and CAP, as well as ethanolic extract, are positioned on the positive side of PC1, while SALA, FGS, and DW extracts are positioned on the negative side of PC1. FGS and DW extracts appear in the lower left quadrant of the PCA score plot due to their low content of phenolic compounds, flavonoids, NDGA, and alkaloids. However, despite their low concentrations of these compounds, they demonstrate a significant ABTS^•+^ scavenging capacity. Finally, the lower right quadrant is represented by the ethanolic extract characterized not only by good antioxidant activity but also by total flavonoid content. In line with the clustering pattern of the metabolomics heatmap, PCA offers a comprehensive knowledge of the relationships and variabilities between *L. cuneifolia* conventional and non-conventional extracts, highlighting its significance in this investigation.

### 2.5. Antibacterial Activity

Recently, it was shown that NADESs might be used not only for their valuable properties as solvents but also to enhance the biological activity of dissolved active compounds. Due to this, we decided to test the antimicrobial activity of the extracts and compare the results to the activity of conventional extracts, based on the total phenolic content.

In this way, a macrodilution assay was used to investigate the antibacterial activity of *L. cuneifolia* extracts against many multidrug-resistant pathogen bacterial strains. Along with the antibacterial activity of the extracts, the activity of pure NADES systems was tested at an applied dilution range for the extract, i.e., pure solvents were applied in a volume equivalent to the concentration used to test the antimicrobial activity of the extracts.

The MICs of tested extracts and pure NADES are shown in Table 3. As can easily be observed, all extracts prepared show higher antibacterial activity against the tested Gram-positive bacteria than the Gram-negative strains used.

In the case of the aqueous extract, it does not show growth inhibition in all bacteria tested at the maximum concentration assayed (=600 µg GAE/mL), nor do the FGS extract and NADES-FGS. In contrast, ethanolic extract shows antibacterial activity in all tested Gram-positive strains with MIC values of 150 µg GAE/mL against *Staphylococcus aureus* and *S. epidermidis* clinical isolates and an MIC value of 75 µg GAE/mL against the reference strains *S. aureus* ATCC 29213, *S. aureus* ATCC 43300, and *Enterococcus faecalis* ATCC 29212. However, ethanolic extract displays no activity against Gram-negative strains at the maximum concentration tested.

The most potent antimicrobial activity was observed for LAS extract against all Gram-positive strains with an MIC equal to 37.5 µg GAE/mL. LAS extract is also active against *Escherichia coli*, *Klebsiella pneumoniae* (MICs are in the range of 75–150 µg GAE/mL), and *Pseudomona aeruginosa* (MIC = 75 µg GAE/mL). However, the pure NADES LAS exhibits growth inhibition with MIC values in the range of 75–150 µg GAE/mL, so the extract antibacterial activity against Gram-positive bacteria can be attributed to a potential synergic effect between the active compounds of *L. cuneifolia* and the NADES components.

CAP extract shows greater activity against Gram-positive strains (75 µg GAE/mL) compared to Gram-negative strains (300 µg GAE/mL). However, no significant difference in MIC values between pure system NADES-CAP and CAP extract for Gram-negative strains is observed. Moreover, pure SALA system and corresponding SALA extracts do not show differences.

**Figure 9:**
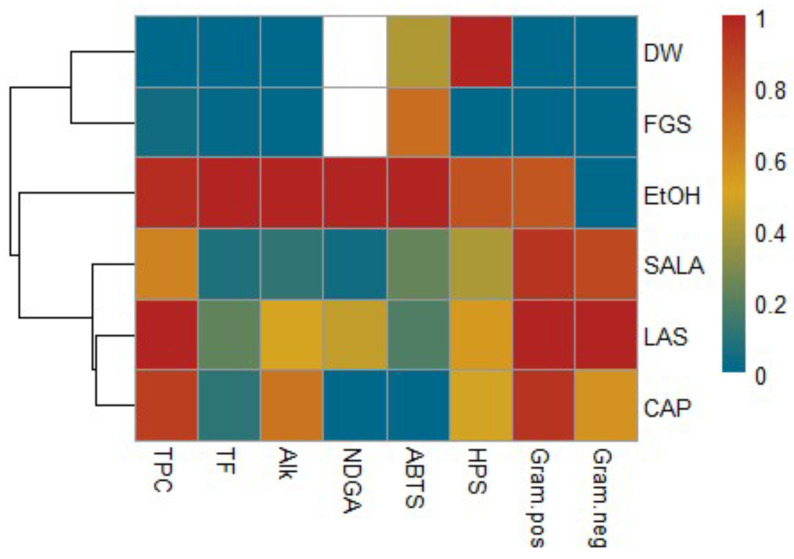
Cluster heatmap analysis summarizing phytochemical characterization and biological activity of *L. cuneifolia* extracts using conventional and non-conventional solvents (NADESs). Columns and rows represent individual variables and different extracts, respectively. Red indicates higher and blue indicates lower metabolite concentration or biological activity. The similarity measurement for clustering was based on the Euclidean distance coefficient. Total phenolic compounds (TPC), total flavonoids (TF), alkaloids content (Alk), NDGA content (NDGA), scavenging capacity of the ABTS radical cation (ABTS), hydrogen peroxide scavenging capacity (HPS), MIC of Gram-positive bacterial strains (Gram.pos), MIC of Gram-negative bacterial strains (Gram.neg), aqueous extract (DW), ethanolic extract (EtOH), lactic acid–saccharose extract (LAS), citric acid–propylene glycol extract (CAP), lactic acid–saccharose–water extract (SALA), fructose–glucose–saccharose–water extract (FGS).

Based on the normalized values of the studied parameters, a heatmap with cluster analysis was created. The heatmap represents the relationship between the different extracts and their phytochemical content as well as their biological activity. The results are visualized with a false color scale with red indicating an increase and blue a decrease in the values of the content of secondary metabolites or biological activity in the different extracts of *L. cuneifolia*. The result of the heatmap analysis of the present study is illustrated in Figure 9. In this study, the extracts are assigned to three groups based on cluster analysis. The first cluster includes the CAP, LAS, and SALA extracts (acid-based NADES systems) according to the HPS capacity, antibacterial activity, and the total phenolic, NDGA, and alkaloid content. The second cluster includes FGS and aqueous extracts. Finally, a separate cluster is formed by ethanolic extraction. Ethanolic extract is distanced from the other extracts, as is also observed in the PCA, due to its good extraction yield and antioxidant activity.

## 3. Discussion

The extraction of phenolic compounds from *L. cuneifolia* using the aforementioned NADESs has received little attention thus far. According to numerous authors, the capacity and selectivity of bioactive compound extraction using NADESs as solvents is closely related to the physicochemical characteristics of the NADES, including polarity, viscosity, and pH [18,20,33]. NADES components are distinguished by the presence of several functional groups such as hydroxyls, carboxyls, or amino groups, which allow for the formation of hydrogen bonds with solutes, thereby significantly increasing the solubility of compounds in NADESs, such as phenolic compounds [21]. Several studies have shown that NADESs improve the extraction efficiency of phenolic compounds, with sugars, alcohols, and organic acids being the most common constituents for their preparation [26,34].

According to our study, NADES systems containing organic acids showed the best extraction efficiency of phenolic compounds from *L. cuneifolia*. It is difficult to select a particular NADES as they have different extraction properties and selectivity. The use of organic-acid-based NADESs, such as LAS and CAP, as a solvent led to a higher extraction performance of total phenolic compounds, considering that both solvents presented similar chemical profiles to EtOH extract. One of the eutectic solvents that achieved the best recovery of NDGA was NADES-LAS, which also exhibited high relative percentages and diversity of NDGA derivatives. These results are promising for the pharmaceutical industry since it is well known that NDGA and its derivatives present numerous medicinal properties such as antioxidant, antibacterial, and anticancer activity [35,36]. Previous studies have reported the extraction of NDGA from *L. cuneifolia* using a NADES composed of lactic acid–dextrose [32]. Furthermore, NADES formulations utilizing organic acids demonstrated superior extraction capabilities for flavonoids, encompassing both glycosides and aglycones, as reported by [37]. However, it is important to take into account that the extraction yield can be influenced not only by the solvent system, but also by the plant matrix.

Flavonoid glycosides are commonly found in plants. The glycosylation of flavonoids depends on factors such as the type and number of sugars (mono-, di-, or tri-) and the type of glycosidic linkage (O- and C-). The NADES-LAS extract exhibited the highest diversity in O-glycosylated flavonoids, whereas DW, EtOH, and SALA extracts predominantly extracted C-glycosylated flavonoids. Glycosylation plays a crucial role in modulating the biological activity and physicochemical properties of flavonoids, enhancing their structural diversity and pharmacological potential. This modification improves the solubility and stability of flavonoid aglycones, which are key factors for their absorption and overall bioavailability. The enzymatic addition of sugar moieties leads to the formation of glycosides, which often display superior pharmacological properties compared to their aglycone counterparts. O-glycosylated flavonoids are more commonly found in nature and exhibit increased water solubility due to the presence of O-glycosidic linkages. This structural feature enhances their bioavailability by overcoming solubility challenges, making them more accessible for biological absorption. The hydrophilic nature of glycosides, resulting from their polar C-O linkages, significantly improves their interaction with water molecules and consequently enhances the pharmacokinetic properties of flavonoids, making them more suitable for pharmaceutical applications. In contrast, C-glycosylated flavonoids exhibit lower water solubility due to the hydrophobic nature of the C-glycosidic bond. This carbon–carbon linkage reduces their solubility compared to their O-glycosylated counterparts, influencing their physicochemical behavior. The inherent hydrophobicity of C-glycosides limits their solubility in aqueous environments, reducing their bioavailability. However, despite their lower solubility, C-glycosylated flavonoids often exhibit enhanced stability and pharmacological properties, which may compensate for their solubility limitations in specific applications [31]. Therefore, the extraction of both O-glycosylated and C-glycosylated flavonoids is of significant interest.

The lignan family exhibits a wide range of pharmacological effects, owing to the extensive structural diversity of lignans that has been discovered. Reported properties include antibacterial, immunosuppressive, anti-asthmatic, antiviral, antioxidant, and anticancer activities [38]. Notably, NDGA, a polyphenol characterized by an o-dihydroxy (catechol) structure, manifests a broad spectrum of biological properties [35]. NDGA presents numerous health benefits related to its strong antioxidant capacity [35] and exhibits antimicrobial activity through synergistic interactions with synthetic antibiotics. Studies have demonstrated that NDGA is able to enhance the efficacy of antimicrobial agents by modulating bacterial membrane permeability and efflux pumps, facilitating drug accumulation at target sites [36]. Furthermore, NDGA has potential as a therapeutic agent for several diseases, including cancer, renal damage, Alzheimer’s disease, and other neurodegenerative disorders [35]. Currently, there are promising anticancer agents derived from NDGA under phase clinical trials, such as terameprocol, commonly known as tetra-O-methyl-nordihydroguaiaretic acid. Although NDGA has been extensively studied over the past few decades, natural NDGA derivatives have been less explored. In this sense, in the extracts of *L. cuneifolia* obtained using NADESs, several natural derivatives of NDGA with similar structures to previously reported synthetic derivatives of NDGA with biological activities were identified. This is an interesting finding as it suggests the potential of harnessing these natural compounds for various biological activities.

Based on current knowledge, alkaloids revealed several biological activities including antibacterial, antioxidant, anti-inflammatory, anti-aging, and anticancer properties [39]. The presence of alkaloids in *L. cuneifolia* was reported for the first time by [32]. These authors quantified theophylline and piperine by HPLC methods using a NADES (lactic acid and dextrose; 5:1) as the extraction solvent. In this study, the total alkaloid content in *L. cuneifolia* extracts varied between 46.15 and 993.84 μg AE/mL, with NADES-CAP emerging as the non-conventional system that demonstrated the best performance. According to the metabolomic analysis, salsolinol and trigonelline hydrochloride were identified. Salsolinol, an isoquinoline alkaloid, was detected in DW, EtOH, and CAP extracts. However, this compound is not exclusive to *L. cuneifolia* but is also found in other plants such as *Aristolochia arcuate*, *Portulaca oleracea*, and *Theobroma cacao*. Numerous studies have reported its diverse biological activities, including neuroprotective effects, antinociceptive activity, and antidepressant properties [40]. Another alkaloid identified in all the extracts was trigonelline hydrochloride, a pyridine alkaloid and derivative of nicotinic acid, which exhibits various biological activities, including hypoglycemic and hypocholesterolemic effects, sedative properties, anti-inflammatory responses, anti-migraine effects, antibacterial and antiviral activities, as well as antioxidant and anti-tumor effects [12]. Therefore, it would be valuable to improve their extraction using NADESs as solvents.

Furthermore, the difference in extraction yields and biological activities for NADES-SALA and NADES-LAS are evident, considering that they are formed by the same constituents (lactic acid and saccharose) but with water as part of the structure of the eutectic mixture (in SALA) and as a diluent of the system (in LAS). Our findings suggest that NADES-LAS displayed better extraction properties for alkaloids and total phenolic compounds, including NDGA and flavonoids. These differences are illustrated in Figure 1 and further highlighted in the heatmap of the metabolomic analysis. Moreover, the *L. cuneifolia* extract using NADES-LAS demonstrated better activity against Gram-positive and Gram-negative strains of medical interest than the extract with NADES-SALA. Regarding the pure solvent, other studies report that the LAS system also inhibits the growth of *S. aureus* [24], consistent with our findings. However, in terms of antioxidant activities, no significant differences were observed between the two systems.

The antioxidant activity of an extract may be due to different mechanisms of action. One of the possible mechanisms of action of antioxidants is by electron or hydrogen atom transfer. In the presence of antioxidant compounds, the transfer of electrons or hydrogen atoms occurs, stabilizing the ABTS radical cation. For the first time, the antioxidant activity of *L. cuneifolia* extracts is reported using NADESs based on sugars, alcohols, and organic acids. However, the choice of a suitable solvent is challenging since all extracts showed antioxidant activity, either by one mechanism of action or another. This behavior was also reported in another study [41]. In our study, the extracts obtained using acid-based systems exhibited the lowest antioxidant activity. This may be due to the low pH of these NADESs, which weakens the proton transfer pathway by suppressing H^+^ ionization and the electron-transfer by the inductive effect [42]. As far as we know, there are few reports on the antioxidant activity from *L. cuneifolia* extracts using organic-acid-based NADESs as a solvent. Refs. [43,44] employed a NADES formed from lactic acid and dextrose (5:1) to extract phenolic compounds from *L. cuneifolia* and reported a similar antioxidant activity between extracts using the NADES in comparison with methanolic extracts.

In addition to the antibacterial activity of the *L. cuneifolia* extracts, the bacterial growth inhibition of pure NADES was studied by testing volumes equivalent to the concentrations assayed for the respective extracts. It was found that organic-acid-based NADESs show antibacterial activity against all tested strains. This activity of pure NADES, such as LAS, CAP, and SALA, can be attributed to them due to their low pH in agreement with other studies [22,45]. Ref. [45] reported that NADESs containing organic acids tested on *E. coli* and *S. aureus* strains produced changes in the environmental pH, altering cell proliferation, metabolism, or denaturing cell wall proteins, ultimately leading to cell collapse and death. Similarly, organic acids such as lactic and citric acid have been widely reported to exhibit antibacterial activity by lowering the pH of their surroundings, thus inhibiting bacterial growth and survival. Studies have shown that even at low concentrations (e.g., 0.03%), citric acid can significantly reduce the growth of *S. aureus*, while lactic acid has been demonstrated to inhibit both *S. aureus* and *E. coli* [46]. These findings emphasize the crucial role of pH reduction as a common mechanism of action in both NADESs and individual organic acids, highlighting their potential as effective antimicrobial agents. Furthermore, our findings confirm the commonly observed trend that Gram-positive bacteria are more vulnerable to antimicrobial agents than Gram-negative bacteria, and this is due to structural differences in their cell walls. The presence of compounds that were identified in *L. cuneifolia* extracts such as naringenin, isorhamnetin, jaceidin, astragalin, limocitrin, piscidic acid, taxifolin, ferulic acid, salsolinol, trigonelline hydrochloride, and lignans would partly explain the antioxidant and antibacterial activity observed. Even though our results demonstrate the ability of *L. cuneifolia* extracts to inhibit bacterial growth, it is important to understand how this antimicrobial activity occurs. Recognizing the mechanisms of action could provide valuable information on the efficacy and selectivity of the extracts, which could contribute to the development of new antibacterial drugs. Therefore, it is suggested that future studies should focus on investigating the mechanisms through which plant extracts exhibit their antimicrobial activity.

To the best of our knowledge, this is the first investigation reporting the extraction of bioactive compounds from *L. cuneifolia* using the previously described eutectic mixtures as sustainable extraction solvents. In addition, several compounds, such as piscidic acid, vicenin-2, yuehgesin, vanillic acid, astragalin, taxifolin, tricin, farrerol, jaceidin, diosmetin, malabaricano, acacetin, and nevandensin, are reported for the first time in this species. Our findings also represent the first report of antioxidant activity in *L. cuneifolia* extracts using these NADES systems. Additionally, this is the first report demonstrating the antimicrobial activity of these extracts against antibiotic-resistant bacterial strains. In this sense, our findings provide additional insight into the potential applications of NADESs to enhance biological activities.

## 4. Materials and Methods

### 4.1. Plant Material

*Larrea cuneifolia* Cav. was collected from Amaicha del Valle (26°36′00″ S 65°55′00″ W; 1960 m a.s.l.) in the Tafí del Valle department, in the northwestern province of Tucumán, Argentina, in summer 2022. Sampling was carried out randomly to form a representative sample of the species. The samples were taxonomically identified by Dr. Soledad Cuello (INBIOFIV-CONICET-UNT). Aerial parts were dried at 40 °C in a forced-air oven then crushed in a grinder and stored at room temperature.

### 4.2. NADES Preparation

Four different NADESs based on organic acids (lactic acid—Cicarelli, Santa Fe, Argentina; citric acid—Merck, Darmstadt, Germany), alcohol (propylene glycol—Biopack, Buenos Aires, Argentina), and sugars (glucose—Anedra, Buenos Aires, Argentina; fructose—Cicarelli, Santa Fe, Argentina; saccharose—Cicarelli, Santa Fe, Argentina) were used. The NADESs were selected based on a literature search, for their different polarities, good viscosity, and density.

The NADESs were prepared by the heating and stirring method [18], since this method shows no differences in the chemical profile to the vacuum evaporation method. Furthermore, this method achieves lower viscosity, resulting in better mass transfer and extraction yield [14]. Briefly, the individual components were weighed out in certain molar ratios and placed in a glass vial with a magnetic stirring bar. The mixture was stirred with heat until a clear, homogenous, and colorless liquid was obtained. The list of synthesized NADESs is shown in Table 1. Due to the high viscosity, a certain percentage of water (% *w*/*w*) was added to some NADESs and stirred for a few more minutes to achieve better solubilization and reduce the viscosity.

### 4.3. Preparation of the Extracts

The extraction of active compounds from *L. cuneifolia* was performed using non-conventional solvents (NADESs) and conventional solvents (70% ethanol and water). Extracts were prepared at a plant/solvent ratio of 1:5 g/mL. The mixture was shaken for 30 min at 100 rpm and 25 °C. Then, it was centrifuged at 10,000 rpm, and the supernatant was collected to obtain the final extracts, which were stored at −20 °C until analysis.

### 4.4. Phytochemical Characterization

The different extracts were standardized according to their content of total phenolic compounds, flavonoids, and alkaloids. Furthermore, the chemical profiles of the extracts were analyzed and compared by thin-layer chromatography (TLC) and ultra-high-performance liquid chromatography (UHPLC-PDA-ESI-QT-MS/MS).

#### 4.4.1. Quantification of Total Phenolic Compounds

The total phenolic content was determined by the Folin–Ciocalteau reagent (Sigma Aldrich, St. Louis, MO, USA) [47]. A solution of gallic acid (1 mg/mL) was used to develop a calibration curve. The measurements were repeated in triplicate using a UV–visible spectrophotometer (JASCO V-630BIO).

The results were expressed as μg gallic acid equivalent (GAE) per milliliter of extract (μg GAE/mL). All NADESs were tested, and a negligible interference in the Folin–Ciocalteu reagent was found even for NADESs which contained sugars.

The stability of the extracts was evaluated by examining the phenolic compound content at two different time points, 3 and 6 months after the extracts were prepared, to determine their stability.

#### 4.4.2. Quantification of Flavonoid Content

The content of total flavonoids was performed according to the method of [48] using AlCl_3_ (Sigma-Aldrich, Darmstadt, Germany). A quercetin solution (1 mg/mL) was used as a reference compound for the construction of a standard curve, and the results were expressed as mg equivalents of quercetin per mL of extract (mg QE/mL extract). Controls were performed for each solvent, and assays were realized in triplicate. Measurements were performed using a UV–visible spectrophotometer (JASCO V-630BIO, Tokyo, Japan).

The flavonoid content of the extracts was measured at two different time points, 3 and 6 months after the extracts were prepared, to determine their stability.

#### 4.4.3. Quantification of Total Alkaloid Content

The total alkaloid content was determined spectrophotometrically according to [49] using bromothymol blue (Sigma Aldrich, St. Louis, MO, USA) as a coloring agent and apomorphine chlorhydrate (Merck, Darmstadt, Germany) as a standard. Total alkaloids were calculated as μg of apomorphine chlorhydrate equivalents per milliliter of extract (μg AE/mL). The conventional and non-conventional solvent controls were performed to determine any possible interference, and assays were performed in triplicate.

#### 4.4.4. Quantification of NDGA: High Performance Liquid Chromatography (HPLC-DAD)

*L. cuneifolia* extracts were analyzed by reverse-phase high-performance liquid chromatography (RP-HPLC) fingerprints. The separation module consisted of a Waters 1525 Binary HPLC Pumps system with a 1500 Series Column Heater, a manual injection valve (Rheodyne Inc., Cotati, CA, USA) with a 20 μL loop and a Waters 2998 photodiode array detector (PDA) (Waters corporation, Milford, MA, USA). An XBridge™ C18 column (4.6 mm × 100 mm, 5 μm; Waters corporation, Milford, MA, USA) with a two-gradient solvent system was used. A system of solvent A (0.1% acetic acid in water) and solvent B (0.1% methanol) (conditions: 10–57% B from 0 to 45 min and 57–100% B from 45 to 65 min) was used. The flow rate was set at 0.5 mL/min. A solution of 3 mg/mL was used. Data collection was carried out with Empower^TM^2 software database version 6.10 (Waters, Milford, MA, USA). Nordihydroguaiaretic acid (NDGA) was identified by comparing its retention time and UV–Vis spectra at 280 nm and quantified using calibration plots that relate peak areas to concentrations of a commercial standard.

#### 4.4.5. Thin-Layer Chromatography

A qualitative technique was performed to compare the chemical profiles of the samples. Equal aliquots of phenolic compounds of each sample were seeded on a silica gel chromatofoil (Kieselgel 60 F254 0.2 mm, Merck). The components were separated using a mobile phase: toluene:chloroform:acetone (4.5:2.5:3.5 *v*/*v/v*).

The separated components were visualized under UV light at 254 nm and 365 nm (Model 5L-58 Mineralight UV Lamp, UVP, Inc., Upland, CA, USA). Phenolic compounds were revealed with NP/PEG reagent (NP 1% diphenylborinic acid aminoethyl ester in methanol, Sigma; PEG: polyethylene glycol and observed under UV light al 365 nm) [50].

#### 4.4.6. Ultra-High-Performance Liquid Chromatography (UHPLC-PDA-ESI-QT-MS/MS)

The separation and identification of the compounds present in the *L. cuneifolia* extracts were performed on a UHPLC-ESI-QTOF-MS system equipped with UHPLC Ultimate 3000 RS with Chromeleon 6.8 software (Dionex GmbH, Idstein, Germany) and Bruker maXis ESI-QTOF-MS with the software Data Analysis 4.0 (all Bruker Daltonik GmbH, Bremen, Germany). For the analysis, the samples were probed to the most efficient dilution for the detection of all metabolites. So, 100 μL of the conventional and non-conventional extracts were diluted in 400 μL of methanol, and 3 µL was injected into the equipment. The chromatographic equipment consisted of a quaternary pump, an autosampler, a thermostatted column compartment and a photodiode array detector. Elution was performed with a binary gradient system with eluent (A) 0.1% formic acid in the water and eluent (B) 0.1% formic acid in the acetonitrile. The gradient was programmed as follows: Solvent A: 88%, decreased to 1% at 15 min, followed by 3 min of isocratic elution with 1% of solvent A and increased to 88% at 18.2 min (total elution time 20 min). Separation was carried out with a Thermo 5 µm C18 80 Å column (150 mm × 4.6 mm) at a flow rate of 0.3 mL/min. ESI-QTOF-MS experiments were recorded in negative and positive ion mode, and the scan range was between 100 and 1200 m/z. Electrospray ionization (ESI) conditions included a capillary temperature of 200 °C, a capillary voltage of 2.0 kV, a dry gas flow rate of 8 L/min, and a nebulizer pressure of 2 bar. The experiments were performed in automatic MS/MS mode. The structural characterization of secondary metabolites was based on HR full MS, fragmentation patterns, and comparisons with the literature data.

### 4.5. Antioxidant Activity

#### 4.5.1. Evaluation of ABTS Radical Cation Scavenging Activity

The free radical scavenging capacity of the different extracts was determined by a spectrophotometric method [51], using the ABTS 2,2-azinobis-(3-ethylbenzothioazolin-6-sulfonic acid) (Sigma Aldrich, St. Louis, MO, USA).

The assay was carried out in a microplate reader (Thermo Scientific Multiskan GO, Vantaa, Finland), and the decrease in the absorbance was recorded at one minute and six minutes after the start of the reaction, and then the percentages of decolorization were calculated. The assays were performed in triplicate. The SC_50_ value (concentration capable of scavenging 50% of the free radicals) was determined for each of the extracts. The antioxidant activity of the pure NADES was also evaluated by testing volumes equivalent to the concentrations assayed for the respective extracts.

#### 4.5.2. Autographic Method for the Detection of Antioxidant Activity Using ABTS^•+^

An autographic assay was performed to detect the antioxidant activity of extracts of complex composition. The chromatographic plates developed as described in Section 4.4.5 were covered with a solution of ABTS^•+^ and 0.9% agar. After solidification, the plate was incubated at room temperature for 1 min in the dark. Antioxidant activity is observed as light spots on a dark blue–green background [52].

#### 4.5.3. Hydrogen Peroxide Scavenging

Hydrogen peroxide scavenging activity (HPS) was evaluated using a reaction mixture containing phenol (12 mM), 4-aminoantipyrine (0.5 mM), hydrogen peroxide (0.7 mM), sodium phosphate buffer at pH = 7 (84 mM), and different concentrations of the extracts. It was kept for 20 min at 35 °C, then horseradish peroxidase (0.1 U/mL) was added, and it was incubated at 37 °C for 30 min [53]. This reaction produces a quinoneimine chromogen that can be measured at 504 nm. The color decrease reflects the hydrogen peroxide scavenged by the plant extracts. The results are expressed as the hydrogen peroxide scavenging percentage (%).

### 4.6. Antibacterial Activity

#### 4.6.1. Microorganisms

Different clinical isolates of multidrug-resistant Gram-positive and Gram-negative bacteria strains were used: *Staphylococcus aureus* (*n* = 10), *Staphylococcus epidermidis* (*n* = 1), *Escherichia coli* (*n* = 6), *Klebsiella pneumoniae* (*n* = 5), and *Pseudomonas aeruginosa* (*n* = 5). Also, the following reference strains were included in the study: *Staphylococcus aureus* ATCC 29213 and ATCC 43300, *Enterococcus faecalis* ATCC 29212, *Escherichia coli* ATCC 35218, *Escherichia coli* ATCC 25922, and *Pseudomonas aeruginosa* ATCC 27853. The clinical samples were obtained from Nestor Kirchner Hospital, San Miguel de Tucumán, Tucumán, Argentina. The *Manual of Clinical Microbiology* [54] was used to identify the bacteria strains. The microorganisms were maintained in Mueller–Hinton (MH) medium supplemented with 30% (*v*/*v*) glycerol at −20 °C. Before testing, the suspensions were transferred to Mueller–Hinton agar and incubated aerobically over night at 37 °C.

#### 4.6.2. Minimum Inhibitory Concentration

MIC values were determined by the macrodilution method in solid medium [55]. The assays were carried out on Mueller–Hinton agar medium. Serial double dilutions of each *L. cuneifolia* extract in a range of 18.25–600 μg GAE/mL were added to an equal volume of medium. The antibacterial activity of the pure NADES was also evaluated by testing volumes equivalent to the concentrations assayed for the respective extracts. Control plates were made containing an equal volume of ethanol or distilled water. After cooling and drying, the plates were spot-inoculated with 2 μL of each bacterial cell suspension (1 × 10^4^ CFU) and incubated aerobically for 18–20 h at 37 °C. A growth control was included for each strain tested. In accordance with the Clinical and Laboratory Standards Institute (CSLI) methodology [55] for the broth macrodilution method, minimum inhibitory concentrations (MICs) of *L. cuneifolia* extracts or solvents used are the lowest concentration of samples at which bacterial growth is not observed after incubation.

### 4.7. Statistical Analysis

Statistical analysis was performed using RStudio version 4.2.1 [56]. The results are the means of three determinations ± standard deviation. Experimental data were analyzed by one-way analysis of variance (ANOVA) applying Tukey’s post-test with a 95% confidence level for all variables, and the significance level was determined (*p* ≤ 0.05). Heatmaps were used to summarize diversity, relative abundance, yield extraction, and antioxidant activity with different solvents. The results were normalized, sorted in ascending order of retention times, and are visualized using a false color scale, with red indicating an increase and blue a decrease in values. No differences are denoted by equal squares. Also, Euclidean distance was used as the similarity measure, and hierarchical clustering with complete linkage was used in the heatmap. Principal component analysis (PCA) was performed on phytochemical content as well as on the antioxidant activity to individuate the most effectively solvent systems. The score plot was performed based on the first and second principal components (PCs). Additionally, a bubble chart was generated to gain further insights into the distribution and abundance of compounds across different extracts.

## 5. Conclusions

In this work, the extraction of bioactive compounds from *L. cuneifolia* using conventional and non-conventional solvents (NADESs) based on organic acids, alcohol, and sugars was reported. NADES systems containing organic acids showed the best extraction efficiency of phenolic compounds from *L. cuneifolia*. The work includes the identification of numerous compounds in the extracts, including phenolic acids, flavonoid glycosides, flavanones, flavones, flavonols, alkaloids, lignans (NDGA and NDGA derivatives), and others, such as piscidic acid, vicenin-2, yuehgesin, vanillic acid, astragalin, taxifolin, tricin, farrerol, jaceidin, diosmetin, malabaricano, acacetin, and nevandensin. All extracts displayed antioxidant potential and antimicrobial activities against clinical isolates of Gram-positive and Gram-negative bacteria and ATCC strains. Heatmap and bubble plot analysis strongly differentiated two groups of extracts (EtOH-LAS-CAP-SALA and DW-FGS) according to the relative abundance and diversity of the identified compounds. Furthermore, the principal component analysis reaffirms this grouping based on the phytochemical characterization and the biological activities evaluated. So, NADESs represent a sustainable alternative for the extraction of bioactive compounds and could therefore replace traditional solvents in the pharmaceutical, cosmetic, or food industry. Further toxicological studies are required to ensure their safe use. This appears to open up new opportunities for a broader application of this plant in the medicinal field.

## Figures and Tables

**Table 2:** Standard deviation, proportion of variance, cumulative proportion, and eigen vectors with respect to the principal components (PC1, PC2, PC3, PC4, PC5, and PC6). Total phenolic compounds (TPC), total flavonoids (TF), NDGA content (NDGA), alkaloid content (Alk), ABTS radical cation scavenging capacity (ABTS^•+^), and hydrogen peroxide scavenging capacity (HPS).

Principal Components	PC1	PC2	PC3	PC4	PC5	PC6
Standard deviation	1.9111	1.1505	0.9425	0.36549	0.04556	3.98 × 10^−16^
Proportion of variance	0.6087	0.2206	0.1480	0.02226	0.00035	0.000
Cumulative proportion	0.6087	0.8293	0.9774	0.99965	1.00000	1.000
Eigen vectors						
TPC	0.4274	0.4807	−0.1538	−0.2068	−0.1560	0.7038
TF	0.4878	−0.3098	−0.0497	−0.0163	0.8105	−0.0794
NDGA	0.5073	0.0818	−0.1036	−0.5558	−0.2326	−0.6016
Alk	0.4923	0.1389	−0.0564	0.8026	−0.2092	−0.2167
ABTS^•+^	0.1777	−0.7976	−0.2167	−0.0286	−0.4533	0.2805
HPS	0.2186	−0.1026	0.9554	−0.0535	−0.1234	0.1030

**Table 3:** MIC values of different extracts and NADESs, against Gram-positive and Gram-negative strains.

Microorganism	DW	EtOH	LAS		CAP		FGS		SALA	
			C	Ext	C	Ext	C	Ext	C	Ext
*S. aureus* (*n* = 10)	>600	150	75	37.5	300	75	>600	>600	75	75
*S. aureus* ATCC 29213	>600	75	75	37.5	300	75	>600	>600	75	75
*S. aureus* ATCC 43300	>600	75	75	37.5	300	75	>600	>600	75	75
*S. epidermidis*	>600	150	75	37.5	300	75	>600	>600	75	75
*E. faecalis* ATCC 29212	>600	150	75	37.5	300	75	>600	>600	75	75
*E. coli* (*n* = 6)	>600	>600	75–150	75	300	300	>600	>600	75–150	150
*E. coli* ATCC 35218	>600	>600	75	75	300	300	>600	>600	75	150
*E. coli* ATCC 25922	>600	>600	75	75	300	300	>600	>600	75	75
*K. pneumoniae* (*n* = 5)	>600	>600	75–150	75	300	300	>600	>600	150	150
*P. aeruginosa* (*n* = 5)	>600	>600	75	75	300	300	>600	>600	75	75
*P. aeruginosa* ATCC 27853	>600	>600	75	75	300	300	>600	>600	75	75

MIC values are the lowest concentration (µg GAE/mL) of samples at which bacterial growth visualized as the formation of a colony is not observed. C: Pure NADES controls. Ext: *L. cuneifolia* extract obtained with the respective NADES.

## Data Availability

The data presented in this study are available on request from the corresponding authors.

## Supplementary Materials

The following supporting information can be downloaded at: https://www.mdpi.com/article/10.3390/plants14071016/s1. Table S1. Tentative identification by UHPLC-PDA-ESI-QT-MS/MS of compounds in *Larrea cuneifolia* Cav. extracts obtained using conventional and non-conventional solvents.
