# Supplementary material for: Extraction of Bioactive Compounds from Larrea cuneifolia Cav. Using Natural Deep Eutectic Solvents: A Contribution to the Plant Green Extract Validation of Its Pharmacological Potential
Source: Plants (Basel). 2025 Mar 24;14(7):1016. doi: 10.3390/plants14071016 (PMC11990737; doi:10.3390/plants14071016)
Supplement: Supplementary file 1 [file plants-14-01016-s001.zip › plants-3532396-supplementary.pdf]

Supplementary material

Table S1. Tentative identification by UHPLC-PDA-ESI-QT-MS/MS of compounds in *Larrea cuneifolia* Cav. extracts obtained using conventional and non-conventional solvents.

Code	Name	RT [min]	m/z meas.	Δm/z [ppm]	M meas.	Ions	Molecular Formula	MS/MS score	Classification	Metabolite type	mSigma	MS/MS fragments
**1**	Trigonelline hydrochloride	0,82	138,05518	2,029	137,0479	[M+H]+	$C_7H_7NO_2$	923,7	Alkaloid	Nicotinic acid derivative	2,2	110.0602, 94.0654, 78.0342
**2**	Salsolinol	0,83	180,10145	-2,504	179,09418	[M+H]+	$C_{10}H_{13}NO_2$	936	Alkaloid	Isoquinoline alkaloid	8,3	163.0762, 145.0652, 117.0732
**3**	D-(-)-quinic acid	0,86	191,05601	-0,526	192,06329	[M-H]-	$C_7H_{12}O_6$	942,4	Other	Carboxilic acid	8,8	93.0345, 85.0293
**4**	Citric acid	0,88	191,01969	-0,191	192,02697	[M-H]-	$C_6H_8O_7$	996,8	Other	Carboxilic acid	12,8	111.0765, 87.00721
**5**	Gentisoyl glucoside	0,89	315,07189	-0,834	316,07917	[M-H]-	$C_{13}H_{16}O_9$	822,3	Phenolic acid	Glycosylated phenolic acid	18,9	153.0165, 87.00721
**6**	Piscidic acid	0,97	255,05126	0,611	256,05854	[M-H]-	$C_{11}H_{12}O_7$	899,3	Phenolic acid		0,7	93.0343, 72.0793
**7**	Demethoxycentaureidin 7-O-rutinoside	0,98	639,21056	29,092	638,20328	[M+H]+	$C_{29}H_{34}O_{16}$	975,8	Flavonoid	O-glycosylated flavonoid	22,1	329.0960, 216.9967
**8**	3,5-Dihydroxy benzoic acid	1	153,01923	-0,693	154,0265	[M-H]-	$C_7H_6O_4$	1000	Phenolic acid		7,4	109.0293, 65.0395
**9**	Vicenin-2	1,01	595,16553	-0,357	594,15826	[M+H]+	$C_{27}H_{30}O_{15}$	904	Flavonoid	C-glycosylated flavonoid	60	271.0602
**10**	Apigenin 6,8-digalactoside	1,04	593,15034	1,267	594,15761	[M-H]-	$C_{27}H_{30}O_{15}$	959,5	Flavonoid	C-glycosylated flavonoid	37,9	473.1032, 383.0792, 353.0672
**11**	Protocatechuic aldehyde	1,11	137,02412	-2,194	138,03139	[M-H]-	$C_7H_6O_3$	867	Phenolic acid		7,7	327.9672
**12**	Yuehgesin	1,13	291,12638	8,88	292,13366	[M-H]-	$C_{16}H_{20}O_5$	947,1	Phenolic acid	Coumarin	28,1	225.0243

**13**	Vanillic acid	1,15	169,05008	3,224	168,0428	[M+H]+	$C_8H_8O_4$	830,8	Phenolic acid		∞	125.0598, 111.0442
**14**	Astragalin	1,18	447,09586	4,014	448,10314	[M-H]-	$C_{21}H_{20}O_{11}$	883,2	Flavonoid	O-glycosylated flavonoid	44,4	284.0341, 255.0311, 227.0355
**15**	Spinacetin 3-gentiobioside	1,33	671,20033	27,618	670,19305	[M+H]+	$C_{29}H_{34}O_{18}$	979,2	Flavonoid	O-glycosylated flavonoid	50,9	345.1237
**16**	p-coumaric acid	1,38	165,05465	0,184	164,04737	[M+H]+, [M-H2O+H]+	$C_9H_8O_3$	865,2	Phenolic acid		10,5	119.0321
**17**	o-coumaric acid	1,43	163,04002	-0,276	164,0473	[M-H]-	$C_9H_8O_3$	933,1	Phenolic acid		11,2	119.0453, 91.0542
**18**	m-coumaric acid	1,64	163,04001	0,369	164,04729	[M-H]-	$C_9H_8O_3$	997,4	Phenolic acid		6,1	119.0508, 93.0347
**19**	Taxifolin	2,2	303,0511	0,555	304,05838	[M-H]-	$C_{15}H_{12}O_7$	941,3	Flavonoid	Flavanone	14,7	258.0485, 213.0548, 153.0179, 123.0445
**20**	Taxifolin isomer	2,46	303,05098	-0,148	304,05826	[M-H]-	$C_{15}H_{12}O_7$	917,7	Flavonoid	Flavanone	12,7	258.0485, 213.0548, 153.0179, 123.0445
**21**	Ferulic acid (4-Hydroxy-3-methoxy cinnamic acid)	2,51	177,0545	-3,07	194,05758	[M-H2O+H]+, [M+H]+	$C_{10}H_{10}O_4$	959,2	Phenolic acid		19,4	107.0089
**22**	Taxifolin	2,74	303,05087	-0,428	304,05814	[M-H]-	$C_{15}H_{12}O_7$	957,5	Flavonoid	Flavanone	23,1	258.0485, 213.0548, 153.0179, 123.0445
**23**	Isoferulic acid (4-4-Methoxy-3-hydroxycinnamic acid)	2,92	193,05059	-0,197	194,05787	[M-H]-	$C_{10}H_{10}O_4$	994,4	Phenolic acid		8,9	133.0263, 42.9979
**24**	Loliolide	3,94	197,1176	1,934	196,11033	[M+H]+	$C_{11}H_{16}O_3$	910,6	Terpen	Monoterpene lactone	7,6	159.00812
**25**	Benzaldehyde	4,21	107,04885	-2,764	106,04157	[M+H]+	C_7H_6O	849,9	Other	Aldehyde	4,6	77.04562

**26**	Dihydrokaempferol	4,64	287,05583	-1,363	288,06311	[M-H]-	$C_{15}H_{12}O_6$	965,7	Flavonoid	Flavanone	9,8	177.0540, 125.0321, 107.0123
**27**	Patuletin 7-glucoside	4,9	493,09908	0,645	494,10636	[M-H]-	$C_{22}H_{22}O_{13}$	916,2	Flavonoid	O-glycosylated flavonoid	18,7	333.0614, 318.0826
**28**	Eriodictyol	5,67	287,05569	-1,477	288,06296	[M-H]-	$C_{15}H_{12}O_6$	985	Flavonoid	Flavanone	15,8	210.5342, 135.0441
**29**	3,9-Dihydroeucomin	5,82	301,10729	0,792	300,10001	[M+H]+	$C_{17}H_{16}O_5$	995,4	Flavonoid	Homoisoflavonoid	15,8	107.0492, 103.0546
**30**	Camelliaside A	5,92	774,26474	25,988	756,23116	[M+NH4]+, [M+Na]+, [M+K]+	$C_{33}H_{40}O_{20}$	983,1	Flavonoid	O-glycosylated flavonoid	6	286.0112, 258.0164, 230.0215
**31**	Quercetin	5,92	301,03531	0,354	302,04258	[M-H]-	$C_{15}H_{10}O_7$	902,1	Flavonoid	Flavonol	5	151.0034, 121.0292
**32**	Isorhamnetin	5,95	315,0511	0,239	316,05827	[M-H]-, [2M-H]-	$C_{16}H_{12}O_7$	907,1	Flavonoid	Flavonol	0,7	299.0166, 151.0035
**33**	Benzaldehyde isomer	5,98	107,04939	2,32	106,04211	[M+H]+	C_7H_6O	849,9	Other	Aldehyde	7,6	77.04563
**34**	Limocitrin	6,16	691,13034	-0,334	346,06872	[2M-H]-, [M-H]-	$C_{17}H_{14}O_8$	879,7	Flavonoid	Flavonol	3,9	315.0076, 287.0202, 258.0146
**35**	Isorhamnetin isomer	6,32	315,05081	-1,348	316,05808	[M-H]-	$C_{16}H_{12}O_7$	977,8	Flavonoid	Flavonol	0,3	299.0166, 151.0035
**36**	5,7,3',4'-Tetrahydroxy-6,8-dimethoxyflavone	6,37	345,06177	0,518	346,06905	[M-H]-	$C_{17}H_{14}O_8$	930,3	Flavonoid	Flavone	23,9	300.0282, 281.8761, 151.0020
**37**	(E)-1,4-bis(4-hydroxy-3-methoxyphenyl)-2,3-dimethylbut-2-ene-1,4-dione	6,49	357,13272	-1,529	356,12544	[M+H]+	$C_{20}H_{20}O_6$	958,4	Lignan	Epoxylignan	21,5	135.0453, 122.0372, 109.2940
**38**	(E)-1,4-bis(4-hydroxy-3-methoxyphenyl)-2,3-dimethylbut-2-ene-1,4-dione	6,51	355,11736	-4,676	356,12464	[M-H]-	$C_{20}H_{20}O_6$	871,8	Lignan	Epoxylignan	21,1	154.0572, 149.0609, 135.0453, 122.0372, 109.2940

**39**	Dihydrobonducellin	6,57	285,11193	-0,733	284,10465	[M+H]+	$C_{17}H_{16}O_4$	928,3	Flavonoid	Homoisoflavonoid	54,7	121.0643, 105.0465, 91.0545
**40**	Naringenin	6,73	271,06107	-0,479	272,06834	[M-H]-	$C_{15}H_{12}O_5$	989,2	Flavonoid	Flavanone	44	187.0417, 151.0058, 119.5253
**41**	Naringenin isomer	6,76	273,07509	-2,428	272,06781	[M+H]+	$C_{15}H_{12}O_5$	978,9	Flavonoid	Flavanone	13,6	153.0177, 119.0492, 91.5431
**42**	Apigenin	6,87	269,04532	-0,762	270,05259	[M-H]-	$C_{15}H_{10}O_5$	976,3	Flavonoid	Flavone	13,4	240.0447, 149.0243, 121.0285, 117.0340
**43**	3,4-Dihydrocoumarin	6,95	149,05961	-0,657	148,05233	[M+H]+	$C_9H_8O_2$	886,1	Phenolic acid	Coumarin	7,1	121.0648, 107.0493, 103.0543
**44**	Benzaldehyde isomer	6,95	107,04929	1,356	106,04201	[M+H]+	C_7H_6O	864,5	Other	Aldehyde	4,6	77.0476, 51.0627
**45**	[4-[3,4,5-trihydroxy-6-(hydroxymethyl) oxan-2-yl] oxyphenyl] methyl 3-acetyloxy-2-hydroxy-2-[(4-hydroxyphenyl) methyl]butanoate	6,95	535,18077	-2,476	536,18805	[M-H]-	$C_{26}H_{32}O_{12}$	840,8	Other		42,4	122.03767, 109.0294
**46**	Phenylacetic acid	7,03	137,05963	-0,465	136,05235	[M+H]+	$C_8H_8O_2$	891,6	Other	Carboxilic acid	6,2	126.1533, 122.1452, 91.1551
**47**	Farrerol	7,05	299,092	-2,457	300,09919	[M-H]-, [2M-H]-	$C_{17}H_{16}O_5$	829,1	Flavonoid	Flavanone	0,7	179.0346, 119.0497
**48**	Tricin (3',5'-di-O-methyltricetin)	7,06	331,08155	0,97	330,07427	[M+H]+	$C_{17}H_{14}O_7$	951,1	Flavonoid	Flavone	∞	299.0165, 271.0256
**49**	Phenylacetaldehyde	7,08	121,06482	0,256	120,05755	[M+H]+	C_8H_8O	927,5	Other	Aldehyde	3,2	91.0238, 77.0645
**50**	Diosmetin	7,09	299,05555	-1,863	300,06283	[M-H]-	$C_{16}H_{12}O_6$	1000	Flavonoid	Flavone	∞	284.0315, 227.0378, 151.0015

**51**	5,7,3',4'-Tetrahydroxy-6,8-dimethoxyflavone isomer	7,18	345,06175	0,465	346,06903	[M-H]-	$C_{17}H_{14}O_8$	778	Flavonoid	Flavone	2,9	300.0282, 281.8761, 151.0020
**52**	Isorhamnetin	7,19	315,0508	-0,644	316,05808	[M-H]-	$C_{16}H_{12}O_7$	975,4	Flavonoid	Flavonol	1,9	300.0282, 281.8761, 151.0020
**53**	Isorhamnetin	7,23	317,06548	0,046	316,0582	[M+H]+	$C_{16}H_{12}O_7$	760	Flavonoid	Flavonol	10,7	300.0282, 281.8761, 151.0020
**54**	Isoferulic acid isomer (2-Hydroxy-4-methoxycinnamic acid)	7,23	193,05052	-0,566	194,0578	[M-H]-	$C_{10}H_{10}O_4$	991,6	Phenolic acid		∞	181.8765, 160.8452, 144.4465
**55**	Irigenin (5,7-dihydroxy-3-(3-hydroxy-4,5-dimethoxyphenyl)-6-methoxychromen-4-one)	7,23	359,07719	-0,154	360,08446	[M-H]-	$C_{18}H_{16}O_8$	779,2	Flavonoid	Isoflavone	3,5	329.0185, 297.0057, 286.0069, 258.0154
**56**	Phenylacetic acid isomer	7,24	137,05985	1,852	136,05258	[M+H]+	$C_8H_8O_2$	986,3	Other	Carboxilic acid	6	126.1532, 122.1454, 91.1547
**57**	Isokaempferide	7,37	299,05603	-0,424	300,06331	[M-H]-	$C_{16}H_{12}O_6$	966	Flavonoid	Flavone	1,9	255.0234, 227.0349, 183.0448
**58**	Jaceidin (Quercetagetin 3,3',6-trimethyl ether)	7,47	359,07701	-0,655	360,08428	[M-H]-	$C_{18}H_{16}O_8$	962,3	Flavonoid	Flavone	2,6	286.0112, 258.0165, 230.0215
**59**	Jaceidin (Quercetagetin 3,3',6-trimethyl ether)	7,51	361,09209	0,812	360,08481	[M+H]+	$C_{18}H_{16}O_8$	760,2	Flavonoid	Flavone	1,8	286.0112, 258.0165, 230.0215

**60**	Quercetagetin 3, 6, 4' trimethyl ether	7,57	359,07709	-0,368	360,08436	[M-H]-	$C_{18}H_{16}O_8$	972,7	Flavonoid	Flavone	3,2	286.0112, 258.0165, 230.0215
**61**	Tricin	7,57	329,06653	-0,527	330,07381	[M-H]-	$C_{17}H_{14}O_7$	962,3	Flavonoid	Flavone	0,4	315.0462, 299.0166
**62**	Quercetagetin 3, 3', 4' trimethyl ether	7,62	361,09217	0,577	360,08489	[M+H]+	$C_{18}H_{16}O_8$	770,5	Flavonoid	Flavone	4,5	286.0112, 258.0165, 230.0215
**63**	Gibberelin A7	7,7	659,28609	-0,126	330,14666	[2M-H]-, [M+HCOOH-H]-	$C_{19}H_{22}O_5$	802,6	Other		22,1	345.1333, 239.1436
**64**	5-(3,4-dihydroxyphenyl)-6,7-dimethyl-5,6,7,8-tetrahydronaphthalene-2,3-diol	7,77	599,26508	-0,432	300,13605	[2M-H]-, [M+Cl]-, [M-H]-, [M+HCOOH-H]-	$C_{18}H_{20}O_4$	975,6	Lignan	Cyclolignan	19,3	135.0453, 122.0372, 109.2940
**65**	Kaempferide	8	299,05585	-0,883	300,06312	[M-H]-	$C_{16}H_{12}O_6$	881,3	Flavonoid	Flavonol	37,1	255.0234, 227.0349, 183.0448
**66**	Phenylacetaldehyde isomer 1	8,04	121,06488	0,743	120,0576	[M+H]+	C_8H_8O	899,3	Other	Aldehyde	3,2	91.0273, 77.0678
**67**	Phenylacetaldehyde isomer 2	8,24	121,06491	1,007	120,05764	[M+H]+	C_8H_8O	885,9	Other	Aldehyde	3,2	91.0275, 77.0672
**68**	Nordihydroguaiaretic acid (NDGA isomer 1, 4-[4-(3,4-dihydroxyphenyl)-2,3-dimethylbutyl] benzene-1,2-diol)	8,25	603,29782	0,601	302,15228	[2M-H]-, [M-H]-	$C_{18}H_{22}O_4$	985,1	Lignan	Epoxylignan	3,6	154.0572, 149.0609, 135.0453, 122.0372, 109.2940
**69**	Nordihydroguaiaretic acid (NDGA isomer 2, 4-[4-(3,4-dihydroxyphenyl)-	8,28	303,15908	0,112	302,15182	[M+H]+, [M+NH4]+, [M+Na]+	$C_{18}H_{22}O_4$	992,7	Lignan	Epoxylignan	2,1	154.0572, 150.0609, 135.0453, 122.0372, 109.2940

	2,3-dimethylbutyl] benzene-1,3-diol)										
**70**	3,6-Dimethoxyapigenin	8,36	329,06661	0,514	330,07389	[M-H]-	$C_{17}H_{14}O_7$	927	Flavonoid	Flavone	2,3	315.0465, 299.0164
**71**	Nordihydroguaiaretic acid (NDGA isomer 3, 4-[4-(2,3-dihydroxyphenyl)-2,3-dimethylbutyl]benzene-1,2-diol)	8,43	301,14493	1,311	302,15235	[M-H]-, [2M-H]-	$C_{18}H_{22}O_4$	951,5	Lignan	Epoxylignan	2,7	154.0572, 149.0609, 135.0453, 122.0372, 109.2940
**72**	Quercetagetin 6, 3', 4' trimethyl ether	8,64	359,07537	-5,224	360,08264	[M-H]-	$C_{18}H_{16}O_8$	958,8	Flavonoid	Flavone	22,5	286.0112, 258.0165, 230.0215
**73**	Ferulic acid isomer (2-Hydroxy-3-methoxycinnamic acid)	8,67	177,05456	0,59	194,05794	[M-H2O+H]+, [M+H]+	$C_{10}H_{10}O_4$	799,6	Phenolic acid		9,2	117.0310
**74**	Santin (5,7-Dihydroxy-3,6,4'-trimethoxyflavone)	8,7	343,08248	1,299	344,08975	[M-H]-	$C_{18}H_{16}O_7$	750,4	Flavonoid	Flavone	4,2	298.0143, 270.0168, 242.0214, 186.0317
**75**	Skullcapflavone II	8,78	375,10774	0,652	374,10047	[M+H]+	$C_{19}H_{18}O_8$	990,9	Flavonoid	Flavone	12,7	327.0487, 197.0065, 169.0143
**76**	5'-(furan-3-yl)-4a-hydroxy-4,7-dimethylspiro[5,6,7,8a-tetrahydro-1H-naphthalene-8,3'-oxolane]-2,2'-dione	8,84	329,13951	-1,085	330,14679	[M-H]-	$C_{19}H_{22}O_5$	880,8	Other	Diterpenic lactone	30,2	122.0425

**77**	Malabaricano	8,99	362,19605	-0,408	344,16232	[M+NH4]+, [M-H2O+H]+	$C_{20}H_{24}O_5$	833	Lignan	Epoxylignan	1,6	271.2163, 203.1073, 188.0836, 151.0753
**78**	Malabaricano isomer	9,01	345,16957	0,608	344,16204	[M+H]+, [M+Na]+, [M+K]+	$C_{20}H_{24}O_5$	929	Lignan	Epoxylignan	6,2	203.1073, 188.0836, 151.0753
**79**	Acacetin	9,04	283,06024	-3,371	284,06752	[M-H]-	$C_{16}H_{12}O_5$	1000	Flavonoid	Flavone	31,5	268.0367, 239.0358, 151.0031
**80**	Lauryl diethanolamine	9,1	274,27417	0,059	273,2669	[M+H]+	$C_{16}H_{35}NO_2$	971,2	Other		3,3	256.2636, 102.0916
**81**	Kuwanon C	9,15	421,16581	0,363	422,17309	[M-H]-	$C_{25}H_{26}O_6$	996,5	Flavonoid	Prenylated flavone	8,2	309.0388, 299.1276, 283.0212
**82**	Caffeic acid	9,3	163,03888	-0,561	180,04147	[M-H2O+H]+, [M+H]+	$C_9H_8O_4$	821,8	Phenolic acid		6,2	117.0334, 89.0376, 95.0494
**83**	Velutin (5-hydroxy-2-(4-hydroxy-3-methoxyphenyl)-7-methoxychromen-4-one)	9,31	315,08638	-0,68	314,07911	[M+H]+	$C_{17}H_{14}O_6$	982,3	Flavonoid	Flavone	5,5	283.0243, 255.0301, 151.0033
**84**	Heminordihydroguaiaretic acid	9,47	315,16038	0,341	316,16808	[M-H]-, [M+HCOOH-H]-	$C_{19}H_{24}O_4$	910,9	Lignan	Epoxylignan	0,8	301.1452, 122.0357
**85**	Cirsimaritin	9,52	313,07174	0,458	314,07902	[M-H]-	$C_{17}H_{14}O_6$	972,4	Flavonoid	Flavone	2,9	283.0243, 255.0297, 163.0036,
**86**	Nordihydroguaiaretic acid (NDGA isomer 4, 4-[4-(2,3-dihydroxyphenyl)-	9,56	301,1455	3,228	302,15278	[M-H]-	$C_{18}H_{22}O_4$	956,2	Lignan	Epoxylignan	1,9	154.0572, 149.0609, 135.0453, 122.0372, 109.2940

	2,3-dimethylbutyl] benzene-1,3-diol)											
**87**	Heminordihydroguaiaretic acid isomer	9,6	315,16054	0,947	316,16788	[M-H]-, [M+Na-2H]-	$C_{19}H_{24}O_4$	964	Lignan	Epoxylignan	2	301.1452, 122.0357, 154.0572, 149.0609
**88**	Nevadensin	9,72	343,08246	0,93	344,08974	[M-H]-	$C_{18}H_{16}O_7$	944,4	Flavonoid	Flavone	3,7	709.1552, 299.0115, 270.0166
**89**	Pachypodol	9,75	345,09718	1,06	344,08983	[M+H]+, [M+Na]+	$C_{18}H_{16}O_7$	883,5	Flavonoid	Flavone	6,8	330.0725, 315.0497, 287.0747
**90**	[5-[4-(3,4-dihydroxyphenyl)-2,3-dimethylbutyl]-2-hydroxyphenyl] 4-hydroxybenzoate	9,77	421,16604	1,432	422,17332	[M-H]-	$C_{25}H_{26}O_6$	994,8	Lignan	Epoxylignan	4,8	301.1452, 122.0357, 154.0572, 149.0609
**91**	[5-[4-(3,4-dihydroxyphenyl)-2,3-dimethylbutyl]-2-hydroxyphenyl] 4-hydroxybenzoate	9,81	423,18029	0,186	422,17302	[M+H]+	$C_{25}H_{26}O_6$	999,7	Lignan	Epoxylignan	30,3	135.0486, 122.0382, 109.0289
**92**	Cinnamaldehyde	9,91	133,0657	6,834	132,05842	[M+H]+	C_9H_8O	826,5	Other	Aldehyde	7	115.0542, 105.0769, 77.0399
**93**	[5-[4-(3,4-dihydroxyphenyl)-2,3-dimethylbutyl]-2-hydroxyphenyl] 4-hydroxybenzoate	9,98	421,16637	2,084	422,17365	[M-H]-	$C_{25}H_{26}O_6$	869,6	Lignan	Epoxylignan	4,9	301.1452, 122.0357, 154.0572, 149.0609, 109.0287
**94**	[5-[4-(3,4-dihydroxyphenyl)-2,3-dimethylbutyl]-	10,01	423,18016	-0,092	422,17361	[M+H]+, [M+Na]+, [M+NH4]+	$C_{25}H_{26}O_6$	999,7	Lignan	Epoxylignan	3	301.1451, 122.0352, 154.0565, 149.0612, 109.0291

	2-hydroxyphenyl] 3-hydroxybenzoate											
**95**	2-oxo-3-phenylpropanoic acid (phenylpiruvic acid)	10,23	163,04013	0,367	164,0474	[M-H]-	$C_9H_8O_3$	1000	Phenolic acid		∞	119.0508, 93.0347
**96**	Cis p-coumaric acid	10,24	147,04429	1,57	164,04769	[M-H2O+H]+, [M+H]+	$C_9H_8O_3$	900,4	Phenolic acid		12,2	119.0506, 93.0344
**97**	Strobilactone A	10,25	265,14745	12,142	266,15472	[M-H]-	$C_{15}H_{22}O_4$	986,9	Other	Steroid lactone	16	192.9691, 79.0212, 61.0193
**98**	Nordihydroguaiaretic acid (NDGA isomer 5, 4-[4-(3-dihydroxyphenyl)-2,3-dimethylbutyl]benzene-1,2,4-triol)	10,52	301,14415	-1,285	302,15142	[M-H]-	$C_{18}H_{22}O_4$	981,3	Lignan	Epoxylignan	6,2	154.0572, 149.0609, 135.0453, 122.0372, 109.2940
**99**	Nordihydroguaiaretic acid (NDGA isomer 6-4[4-(3-hydroxyphenyl)-2,3-dimethylbutyl]benzene-1,2,3-triol)	10,75	301,14513	1,985	302,15241	[M-H]-	$C_{18}H_{22}O_4$	896,1	Lignan	Epoxylignan	5,2	154.0572, 149.0609, 135.0453, 122.0372, 109.2940
**100**	1-(4-hydroxy-3-methoxyphenyl)-7-phenylheptane-3,5-diol	10,83	331,19045	0,187	330,18317	[M+H]+	$C_{20}H_{26}O_4$	888,6	Other	Diarylheptanoid	29,6	122.0367, 109.2951
**101**	Ferulic acid isomer (2-Hydroxy-4-	11,01	195,06526	0,369	194,05798	[M+H]+	$C_{10}H_{10}O_4$	946,7	Phenolic acid		∞	107.0087

	methoxycinnamic acid)											
**102**	1-(2-hydroxy-3-methoxyphenyl)-7-phenylheptane-3,5-diol	11,07	331,1907	0,957	330,18343	[M+H]+	$C_{20}H_{26}O_4$	855,4	Other	Diarylheptanoid	24	122.0371, 109.2983
**103**	3,6-Dimethoxyapigenin	11,45	329,06634	-1,012	330,07362	[M-H]-	$C_{17}H_{14}O_7$	944,2	Flavonoid	Flavone	26,6	300.0282, 281.8761, 151.0020, 117.0354
**104**	(NDGA isomer 7-4[4-(4-hydroxyphenyl)-2,3-dimethylbutyl]benzene-1,2,3-triol)	11,47	301,14483	0,997	302,15211	[M-H]-	$C_{18}H_{22}O_4$	933,3	Lignan	Epoxylignan	3,3	154.0572, 149.0609, 135.0453, 122.0372, 109.2940
**105**	[5-[4-(2,4-dihydroxyphenyl)-2,3-dimethylbutyl]-2-hydroxyphenyl] 4-hydroxybenzoate	11,62	421,16928	8,59	422,17656	[M-H]-	$C_{25}H_{26}O_6$	967,1	Lignan	Epoxylignan	35,1	135.0453, 122.0372, 109.2940
**106**	Masoprocol (nor-dihydroguaiaretic acid or 4-[4-(3,4-dihydroxyphenyl)-2,3-dimethylbutyl] benzene-1,2-diol)	11,76	301,14518	2,147	302,15246	[M-H]-	$C_{18}H_{22}O_4$	943,5	Lignan	Epoxylignan	4,7	154.0572, 149.0609, 135.0453, 122.0372, 109.2940
**107**	3',4'-di-O-methyltricetin	12,09	329,06698	0,915	330,07425	[M-H]-	$C_{17}H_{14}O_7$	841	Flavonoid	Flavone	∞	315.0462, 299.0166
**108**	Canrenone	12,72	339,19988	9,813	340,20715	[M-H]-	$C_{22}H_{28}O_3$	1000	Other	Steroid lactone	35,4	183.0123
**109**	Linoleic acid	15,37	281,24676	-2,652	280,23958	[M+H]+, [M-H2O+H]+	$C_{18}H_{32}O_2$	763,2	Other		17,5	245.2262

**110**	Pheophorbide A	15,77	593,2764	0,933	592,26912	[M+H]+	$C_{35}H_{36}N_4O_5$	901,5	Other	9,3	615.2574
**111**	Pheophytin A (chlorophyll a)	16,16	871,5712	-2,289	870,56393	[M+H]+	$C_{55}H_{74}N_4O_5$	915,5	Other	∞	615.2558
**112**	Pheophytin A isomer	16,36	871,57701	4,371	870,56973	[M+H]+	$C_{55}H_{74}N_4O_5$	900,3	Other	∞	615.2571
**113**	Erucamide	18,48	338,34173	-0,228	337,33454	[M+H]+, [2M+H]+	$C_{22}H_{43}NO$	850,6	Other	8,3	303.30251, 321.31362
